# Deciphering the *Theobroma cacao* self-incompatibility system: from genomics to diagnostic markers for self-compatibility

**DOI:** 10.1093/jxb/erx293

**Published:** 2017-10-07

**Authors:** Claire Lanaud, Olivier Fouet, Thierry Legavre, Uilson Lopes, Olivier Sounigo, Marie Claire Eyango, Benoit Mermaz, Marcos Ramos Da Silva, Rey Gaston Loor Solorzano, Xavier Argout, Gabor Gyapay, Herman Ebai Ebaiarrey, Kelly Colonges, Christine Sanier, Ronan Rivallan, Géraldine Mastin, Nicholas Cryer, Michel Boccara, Jean-Luc Verdeil, Ives Bruno Efombagn Mousseni, Karina Peres Gramacho, Didier Clément

**Affiliations:** 1 Centre de Cooperation Internationale en Recherche Agronomique pour le Developpement (CIRAD), UMR AGAP, Avenue Agropolis TA, Montpellier Cedex, France; 2 Centro de Pesquisas do Cacau (CEPEC), CEPLAC, Rod. Ilhéus-Itabuna, Ilhéus, BA, Brazil; 3 Centre de Cooperation Internationale en Recherche Agronomique pour le Developpement (CIRAD), UR Bioagresseurs, Elig-Essono, Yaoundé, Cameroun; 4 Institut de Recherche Agricole pour le Developpement (IRAD), Yaoundé, Cameroun; 5 Instituto Nacional de Investigaciones Agropecuarias (INIAP), EET-Pichilingue. CP 24 Km 5 vía Quevedo El Empalme, Quevedo, Los Ríos, Ecuador; 6 Commissariat à l’Energie Antomique (CEA), Institut de Génomique (IG), Genoscope, Evry, France; 7 Mondelez UK R&D Limited, Bournville Place, Bournville Lane, Birmingham, UK

**Keywords:** Candidate genes, diagnostic markers, fine mapping, gene expression, immunolocalization, self-incompatibility, *Theobroma cacao*

## Abstract

Cocoa self-compatibility is an important yield factor and has been described as being controlled by a late gameto-sporophytic system expressed only at the level of the embryo sac. It results in gametic non-fusion and involves several loci. In this work, we identified two loci, located on chromosomes 1 and 4 (CH1 and CH4), involved in cocoa self-incompatibility by two different processes. Both loci are responsible for gametic selection, but only one (the CH4 locus) is involved in the main fruit drop. The CH1 locus acts prior to the gamete fusion step and independently of the CH4 locus. Using fine-mapping and genome-wide association studies, we focused analyses on restricted regions and identified candidate genes. Some of them showed a differential expression between incompatible and compatible reactions. Immunolocalization experiments provided evidence of CH1 candidate genes expressed in ovule and style tissues. Highly polymorphic simple sequence repeat (SSR) diagnostic markers were designed in the CH4 region that had been identified by fine-mapping. They are characterized by a strong linkage disequilibrium with incompatibility alleles, thus allowing the development of efficient diagnostic markers predicting self-compatibility and fruit setting according to the presence of specific alleles or genotypes. SSR alleles specific to self-compatible Amelonado and Criollo varieties were also identified, thus allowing screening for self-compatible plants in cocoa populations.

## Teaser

Two different mechanisms controlling the *Theobroma cacao* self-incompatibility system were identified at two separate loci together with candidate genes differentially expressed between self-compatible/incompatible reactions, and diagnostic markers predicting self-compatible varieties were produced.

## Introduction

*Theobroma cacao* L. is a tropical tree originating from the humid forests of the northern part of South America, where large genetically diverse populations still exist *in situ* (Cheesman, 1944; Lanaud, 1987; Laurent *et al.*, 1993, 1994; Figueira *et al.*, 1994; Motamayor *et al.*, 2002, 2008; Loor Solorzano *et al.*, 2012). *Theobroma cacao* seeds are used to make chocolate, providing income for at least five million smallholders worldwide. Smallholder farmers typically cultivate between one and five hectares, achieving low yields. One important yield factor is the self-compatibility status of cocoa trees. Self-compatible (SC) young trees would produce an average of 66% more fruits than the self-incompatible (SI) trees (Cope, 1939*a*), and a higher proportion of self-compatible trees has been observed among higher-producing trees (Lachenaud *et al.*, 2005). Various mechanisms of self-incompatibility (SI), preventing the self-fertilisation of plants and thus favouring plant heterozygosity, have been described in angiosperms (de Nettancourt, 1997; Takayama and Isogai, 2005; Rea and Nasrallah, 2008). These mechanisms act at the level of inhibition of pollen growth for most species, or at the ovary level, leading to a failure of embryo development. Several mechanisms of genetic control of SI have been described in plants, as follows.

The gametophytic S-RNase-based self-incompatibility system (GSI), which has been described in Solanaceae, Rosaceae, and Plantaginaceae, is controlled by a multi-allelic S-locus that includes at least two genes whose interaction will lead to pollen rejection. The female determinant codes for an extracellular ribonuclease (S-RNAse) and the male determinant codes for a protein containing the F-box motif SFB (*S* haplotype-specific F-box protein) (McClure, 2008; Sassa *et al.*, 2010; McClure *et al.*, 2011). The pollen is rejected when there is a match between the single S-haplotype in the haploid pollen and either of the two S-haplotypes in the diploid pistil.

The sporophytic self-incompatibility system (SSI), which has been well described in Brassicaceae, is controlled by one highly polymorphic S-locus that contains two tightly linked genes: the S-locus Cys-rich (SP11/SCR) gene, which is the male determinant, producing proteins that are secreted from the anther tapetum and localized at the surface of pollen grains (Schopfer *et al.*, 1999; Suzuki *et al.*, 1999), and the stigma S-locus receptor Ser/Thr kinase (SRK), which is the female determinant, localized in the stigma cells (Stein *et al.*, 1991). During incompatible reactions, the self-incompatibility response is mediated by allele-specific interactions of the SP11/SCR and SRK genes. In the SSI system, the SI phenotype of the pollen is determined by its parental genotype where dominance relationships exist between alleles, and both possible pollen alleles express the same SI phenotype when interacting with the stigma cells.

A late-acting self-incompatibility system (LSI) has been also described in several species (for reviews see Seavey and Bawa, 1986; Gibbs, 2014). The SI mechanism in *T. cacao* has been described as a LSI system: in SC and SI reactions the pollen can reach the ovary with a similar rate of pollen-tube growth 4 h after pollination (Bouharmont, 1960), but the double-fertilization is completed after 24 h in SC reactions, whereas in SI reactions the male nuclei are released into the embryo sac, but fail to achieve gamete fusion in part of the ovules, resulting in floral abscission (Cheesman, 1927; Cope, 1939*b*, 1940, 1958, 1962; Posnette, 1940; Knight and Rogers, 1955; Bouharmont, 1960; Ford and Wilkinson, 2012). The genetic control of *T. cacao* SI has been studied by several authors (Cope, 1939*b*, 1940, 1958, 1962; Knight and Rogers, 1955; Glendinning, 1967), who hypothesized the existence of an S-locus and several alleles with dominance relationships between them, including an amorphous *Sf* allele that is present in the SC variety Amelonado, and leading to self-compatibility when homozygous. Several authors have contributed to establishing the dominance relationships between the S alleles. Knight and Rogers (1955) first established the relationships between five S-alleles: S1>S2=S3>S4>S5. Cope (1962) and Glendinning (1967) found additional alleles, and determined that S4 and S5 are recessive to the Amelonado self-compatible allele (*Sf*). The dominance relationships between all alleles were established as S0=S1>S2=S3>*Sf*>S4>S5.

 In addition, two other loci involved in *T. cacao* self-incompatibility were also hypothesized (Cope, 1962), based on cytological studies showing 25%, 50%, or 100% of ovules without gamete fusion after selfing of SI cocoa trees. It was concluded that the genetic system of SI in cacao has both aspects of sporophytic and gametophytic control (Cope, 1958). Indeed, the success of gamete fusion depends to the interactions between specific male and female alleles, as in a gametophytic system, but it also depends on the interactions between alleles in the parental tissues, as in a sporophytic system. This leads to dominance relationships between the two alleles that either allow or prevent the activation of the incompatibility reaction.

Two loci putatively involved in self-incompatibility as determined by the percentage of fruit setting have been localized by QTL analysis: one is localised at the top of chromosome 4 (Crouzillat *et al.*, 1996; Royaert *et al.*, 2010; Yamada *et al.*, 2010), the other in chromosome 7 (Yamada *et al.*, 2010). Failure of gamete fusion, observed in part of the ovules and associated with fruit abscission, was observed only at the embryo-sac level (Cope, 1939*b*; Bouharmont, 1960). However, fruits can reach maturity after self-pollination of a SI tree when compatible pollen is mixed with pollen from the SI trees (the ‘mentor pollen’ effect) (Opeke and Jacob, 1967; Bartley, 1969, 2005; Lanaud *et al.*, 1987; Glendinning, 1960), allowing the development of fertilized ovules. With the help of molecular markers, the observation of skewed segregations potentially linked to SI is then possible among the progenies.

Here, we report the analysis of a large F_2_ progeny produced from a SI tree using the ‘mentor pollen’ effect. A first molecular analysis, performed on a subsample of this F_2_ progeny, revealed a skewed segregation at the level of CH1 and CH4 regions (Allegre *et al.*, 2012). In this current work, we carried out fine mapping of these genome regions and identified candidate genes whose expression was then characterized during SC and SI reactions. In order to refine the existing predictive model for self-compatibility (Da Silva *et al.*, 2016), we searched for multi-allelic diagnostic simple sequence repeat (SSR) markers in the CH4 region identified by fine mapping.

## Materials and methods

### Population used for fine mapping

A large progeny was produced by CEPLAC, at Itabuna (Brazil), by selfing the clone TSH516, a hybrid of ICS 1 and Scavina 6 (SCA 6). ICS 1 is a SC Trinitario clone selected in Trinidad, a hybrid between Criollo and Amelonado (two SC varieties). SCA 6 is a SI Upper Amazon Forastero clone collected in Peru. In this study, a total of 877 individuals from this progeny were analysed.

TSH516 is a SI clone, but self-seeds were produced with mixed pollinations using pollen from *Herrania mariae* (closely related to the *Theobroma* genus) as mentor pollen, followed by pollination with TSH516 self-pollen, as described previously (Bartley, 1969, 2005; Opeke and Jacob, 1967). Only TSH516 self-fertilized seeds developed into mature seeds while embryos from the interspecific cross were aborted (flat seeds). The resulting progeny was considered as an F_2_ in this experiment, although TSH 516 parents are not pure lines.

### Analyses of potential skewed segregations in other progenies

A progeny of 550 individuals was created at IRAD (Barombi-kang, Cameroon) from IMC 60, a SI clone originating from Peru. Pollinations were made using a mixture of pollens of IMC 60 and of Catongo (a SC Amelonado-type originating from Brazil) as mentor pollen. Self progenies were identified using molecular markers.

Another progeny of 96 plants, UF 676×ICS 95, planted in French Guyana, was analysed to observe the segregations in the CH1 locus: UF 676 (SI) and ICS 95 (SC) are two hybrid Trinitario plants, heterozygous for an Amelonado allele and a Criollo allele for the CH1 locus. This cross is compatible.

### Populations used for a genome-wide association study (GWAS) and to analyse the prediction efficiency of diagnostic markers

A population of 710 individuals from different origins was used to assess the efficiency of prediction based on SSR genotypes and alleles. This population was made up as follows: 151 individuals belonging to several commercial varieties (full-sib progenies) selected within a cocoa farm in Cameroon; 125 individuals from a breeding population from IRAD; 270 individuals from a breeding population from CEPLAC; 97 individuals from a collection of Nacional-type accessions from INIAP, resulting from germplasm conservation or new breeding steps; nine other accessions from Upper or Lower Amazon regions from CIRAD; and 58 individuals from a germplasm collection of Trinitario- and hybrid-types from the CRC (Trinidad and Tobago), previously evaluated for SC (Bartley, 1970) and stored in the ICGD database (http://www.icgd.reading.ac.uk).

For determination of the SC status of individuals not previously evaluated, 30 to 60 floral buds were covered one day prior to hand-pollination, and for a 3-d period after pollination. Half of the flowers were pollinated using pollen from the same tree and the other half were pollinated using control SC pollen. The percentage of setting was observed after a 14-d period; trees showing less than 10% fruit set after self-pollination and more than 30% after cross-pollination were considered as SI.

A subset of 570 individuals from the populations was analysed by GWAS using single nucleotide polymorphism (SNP) markers.

### Samples for gene expression analyses

Pollinations were carried out on the SCA 6 SI clone in the greenhouse of CIRAD/Montpellier using pollen from ICS 1 (SC) or from SCA 6 (SI), and ovaries with their pistil (termed simply ‘ovaries’ in the text) were collected at different times after pollination. RNA was extracted from unpollinated ovaries and from ovaries collected at 2, 5, 8, 12, 24, 48, and 72 h after SC and SI pollinations. RNA was pooled for samples taken at 2–8 h, 12–24 h, and 48–78 h after pollination. Quantitative PCR (qPCR) was conducted on three biological repetitions of each RNA extract.

### Genotyping

#### F_2_ progeny

Our objective was to identify skewed regions with a total absence of recombinant plants, linked to the SI reaction, in the CH1 and CH4 regions of the F_2_ progeny. Several approaches were used to define new markers in these two regions.

SSR markers were defined using the whole sequence of the Criollo genome V1 (http://cocoa-genome-hub.southgreen.fr/gbrowse) (Argout *et al.*, 2011), and the tool ‘Search for SSR’ in the ESTTIK database (http://esttik.cirad.fr/cgi-bin/SSR_server.cgi) (Supplementary Table S1 at *JXB* online). PCR amplifications and genotyping of the different populations were performed according to Loor Solorzano *et al.* (2012).

Two different strategies were used to identify deletions/insertions. First, deletions/insertions were identified in the CH4 region between the Criollo genome and the Amelonado genome (http://www.cacaogenomedb.org/) (Motamayor *et al.*, 2013) after alignment of both sequence fragments using ClustalW2 implemented at the SimGene.com website (http://simgene.com/ClustalW). Second, resequencing of the CH1 and CH4 regions was made by DNA capture in 24 accessions of different genetic origins. Capture oligos were designed by Roche NimbleGen using the targeted regions of the reference sequence of Criollo genome V1. The captures were carried out according to the protocol provided by NimbleGen (SeqCap EZ Library SR—User’s Guide version 4.0).

Primers amplifying fragments including the deletion were defined using the primer-BLAST online tool from NCBI (http://www.ncbi.nlm.nih.gov/tools/primer-blast) and checked for their polymorphism in the F_2_ progeny. Newly designed markers were named mSI_X. The mTcCIRx markers used in this study can be found in the TropGENE database (http://www.southgreen.fr/content/tropgene-database).

#### Seedlings obtained from mixed pollinations performed on IMC 60

We used four SSR markers to differentiate seedlings produced from self- or cross-fertilization within the IMC 60 progeny after mixed pollinations: mSI_303, mSI_458, mSI_460, and mTcCIR_7, discriminating IMC 60 from Catongo.

#### GWAS population

The GWAS population was genotyped by sequencing (GBS) using the DArTseq technology after DNA restriction with *Pst*I and *Mse*I (Kilian *et al.*, 2012).

### Cytological observation of the proportion of fertilized ovules

Several SC trees from the F_2_ progeny, homozygous for the Amelonado allele for the CH4 locus and heterozygous for the CH1 locus, were self-pollinated or pollinated with a SC pollen (CCN 51) taken as a control. Young fruits were harvested 7 d after self-pollination and to determine the proportion of fertilized ovules, the fruits were boiled for 20 min in water and then dissected. Using a binocular microscope, the relatively large, white fertilized ovules could be distinguished from the poorly developed, yellow, aborted ovules.

### Genome-wide association studies

GWAS was conducted using Tassel 5.2.31 software (Glaubitz *et al.*, 2014) on 570 individuals assessed for SC/SI and coded as 0 (SI) and 1 (SC). In addition, for a subsample of 388 individuals the percentage of fruit setting 14 d after self-pollination was determined as a quantitative variable to assess SC/SI status. The 570 individuals were genotyped by sequencing using 16480 SNPs stored in the TropGENE database (http://tropgenedb.cirad.fr/tropgene/) and with a minor allele frequency >0,05. The mixed linear model method was applied to identify marker/trait association using the population structure and kinship matrix as covariates (Bradbury *et al.*, 2007). The structure of the population was determined with a subset of 150 SNPs distributed over all chromosomes, and using a Bayesian clustering method implemented in the STRUCTURE software (Pritchard *et al.*, 2000), with a burning period of 100 000 iterations, 500 000 Markov Chain Monte Carlo repetitions, and 10 independent runs. The variances of LnP(D) (Estimated Ln Prob of Data) were checked for each run, and runs for which the variance was not homogeneous with variances of the other runs with the same *K* value were eliminated.

A model with admixture and correlated allele frequencies was chosen, and with a *K* number of sub-populations between 5 and 12. The value of *K* was estimated as in Evanno *et al.* (2005). The contributions of individuals to the *K* groups were then used as covariates in the analysis. The kinship matrix was constructed from the 16480 SNPs.

Significant associations between markers and SC/SI status were identified in two steps: first, the *P* values were computed, and second, to eliminate false-positive associations, the *P*-value threshold was determined using the QVALUE software to control the false discovery rate (FDR) (Storey, 2002). The corresponding *P*-value threshold was given by the QVALUE with a cut-off for rejection of the null hypothesis (*q* value) of 0.05 to ensure a 5% FDR.

### Search for candidate genes

The Criollo cocoa genome sequence, version V1, available at the Cocoa Genome Hub (http://cocoa-genome-hub.southgreen.fr/gbrowse) (Argout *et al.*, 2011), was used to identify candidate genes involved in the *T. cacao* SI system, on the basis of their homology with genes already known to be involved in SI systems in other species. Each ortholog to *T. cacao* genes annotated in the Criollo genome for the CH1 and CH4 genome regions without recombinant plants was checked for its potential role in self-incompatibility systems already described in the literature.

### Gene expression analyses

The expression of candidate genes identified in the CH1 and CH4 regions was analysed in the SCA 6 accession during SC and SI reactions.

Ovaries were harvested at various time intervals after SC and SI pollinations (0 h; 2-5-8 h; 12-24 h, and 48-72 h) and conserved at –20 °C in RNA*later* solution (Sigma). Isolation of total RNA and cDNA synthesis were conducted according to Legavre *et al.* (2015).

Gene expression was assessed by qPCR using a Roche LightCycler 480 Real-time PCR System with a SYBR Green dye included in the supermix to detect dsDNA amplification products. qPCR was carried on 12 candidate genes together with two reference genes that were used for normalization, Tc04_g000050 (Isocitrate dehydrogenase) and Tc08_g003640 (Tubulin beta-6 chain), as previously described (Pinheiro *et al.*, 2011). Primers were designed using the Primer3 program (http://bioinfo.ut.ee/primer3-0.4.0/) and qPCR was performed as described in Legavre *et al.* (2015) (see Supplementary Table S2). The absence of non-specific products was verified by a melting-curve analysis.

The amplification efficiency of each primer pair was determined using a standard procedure and integrated in the analyses. Differential expression of each candidate gene was calculated according to the 2^–∆∆Ct^ method (Livak and Schmittgen, 2001). After normalization, the relative expression of candidate genes was analysed in relation to the unpollinated ovaries.

### Immunolocalization of Tc01_g007270 and Tc01_g007290 proteins

Immunostaining was performed on 8-μm paraffin sections of unpollinated ovaries and SC-pollinated or SI-pollinated ovaries (8–24 h) after fixation in 3% paraformaldehyde/0.1% Triton X-100 in phosphate buffer and embedding in paraplast blocks (Brillouet *et al.*, 2014).

Rabbit polyclonal antibodies anti-Tc01_g007270 and anti-Tc01_g007290 were produced by Eurogentec (Anti-peptide Speedy 28-Day, https://secure.eurogentec.com/speedy.html) using synthesized peptides as the antigen. The peptide sequence LGNDKTVRIWTQENE corresponds to residues 310–324 of the Tc01_g007270 protein and RSVDKSNDESESQVS corresponds to residues 478–492 of the Tc01_g007290 protein. Optimal antibody dilutions were determined by ELISA tests (final dilutions: 1:500 for anti-Tc01_g007270 and anti-Tc01_g007290, 1:200 for pre-immune serums). Controls were carried out with pre-immune serum. Immunolocalization was determined with an Alexa Fluor 488 dye conjugated goat anti-rabbit antibodies (Interchim, France, Montluçon).

Microscopic imaging was performed using the Montpellier RIO Imaging Platform (https://www.mri.cnrs.fr/fr/) with a confocal microscope (LSM510, Meta; Carl Zeiss Micro Imaging) or with a NIKON Eclipse Ni-E light microscope using the filters DAPI (340–380/400) and B2 A (450–490/505) for fluorescence.

### Identification of diagnostic markers

To determine diagnostic markers, 11 SSR markers, identified in the CH1 and CH4 regions, were used to genotype the full population of 710 individuals, and to establish predictions for SC/SI according to the genotype or allele. Each marker was either considered alone or in combination with one or two other markers.

#### Allelic frequencies and identification of alleles specific to Amelonado and Criollo SC varieties

A set of 108 genotypes, capturing the diversity of the *T. cacao* genetic groups and planted in the international collection of the CRC, were genotyped with the 11 SSR markers. Allele frequencies were estimated for each genetic group, using the GENETIX software (http://www.genetix.univ-montp2.fr/genetix/constr.htm) (Belkhir *et al.*, 2004), and a search was made for alleles specific to the SC varieties Amelonado and Criollo. Reference SSR profiles allowing cocoa breeders to characterize clones for potential SC/SI status were established for these SSRs (see Supplementary Table S3).

Prediction analyses were carried out according to genotype or allele presence, using SAS software modules (SAS Institute Inc., 2004):

#### Genotype analyses

All combinations were tested by Fisher’s Exact Test, using PROC FREQ. Probabilities associated to the tests were adjusted for multiple testing, using the QVALUE software (Storey, 2002). The probabilities of being SC given the individuals have (ProbSIYs) or do not have (ProbSINo) the specified genotype were computed from a logistic regression computed through PROC LOGISTIC. Here, we tested the effects of all 36 939 combinations of genotypes (1 to 3 loci) in the self-incompatibility.

#### Allele analyses

With the aim of verifying if the presence of a given allele has a significant effect on the proportion of SC/SI plants, the allele effect was tested for each of the 128 alleles by Fisher’s Exact Test, using PROC FREQ, considering that in the present case many cells had small expected counts. Probabilities associated to the tests were adjusted for multiple testing, using QVALUE. The effect of increasing the allele dose was evaluated by fitting a logistic regression to the data, using PROC LOGISTIC. The probabilities of being SC/SI when the individual has 0, 1, or 2 copies were estimated.

## Results

### Study of skewed segregations linked to the incompatibility system in the F2 progeny

Fine-mapping of restricted genome regions was conducted, including the skewed segregations in the F2 progeny. The alleles of TSH516 inherited from ICS 1 in both the CH1 and CH4 regions are ‘Amelonado alleles’. In these regions, three possible genotypes were expected: homozygous for the Amelonado allele (a), homozygous for the allele provided by SCA 6 (b), and heterozygous (h).

In the skewed CH1 region, 22 markers could be defined and were used to genotype the F_2_ progeny. Among the three possible types, the b and h genotypes were predominant in this region, with the ‘a’ Amelonado genotype totally absent in a small region of 70.3 kb delimited by the markers mSI_103 and mSI_375. A total of 761 individuals were analysed for some markers of this region (Table 1).

**Table 1.** Segregations observed in the F_2_ progeny at the level of the CH1 and CH4 genome regions

Markers	Chromosome	Position (bp)	Number of individuals			
			a	h	b	Total
mSI_26	CH1	3 377 732	5	228	118	351
mSI_88	CH1	3 499 444	5	224	111	340
mSI_89	CH1	3 525 756	5	233	114	352
mSI_32	CH1	3 649 333	4	236	120	361
mTcCIR15	CH1	3 711 664	4	227	117	348
mSI_73	CH1	3 790 637	4	291	138	429
mSI_101	CH1	3 935 902	3	243	119	365
mSI_102	CH1	3 966 163	2	249	119	370
mSI_140	CH1	3 988 656	2	217	125	344
mSI_141	CH1	4 010 921	2	308	138	448
mSI_103	CH1	4 021 267	3	387	228	618
**mSI_366**	**CH1**	**4 053 385**	**0**	**508**	**253**	**761**
**mSI_367**	**CH1**	**4 054 418**	**0**	**366**	**216**	**582**
**mSI_369**	**CH1**	**4 057 532**	**0**	**419**	**221**	**640**
**mSI_440**	**CH1**	**4 066 036**	**0**	**476**	**253**	**729**
**mSI_370**	**CH1**	**4 070 474**	**0**	**466**	**266**	**732**
**mSI_372**	**CH1**	**4 073 585**	**0**	**297**	**170**	**467**
mSI_375	CH1	4 091 577	2	429	228	659
mSI_107	CH1	4 130 575	4	374	212	590
mTcCIR356	CH1	4 149 062	6	232	118	354
mSI_112	CH1	4 233 257	6	339	163	502
mSI_113	CH1	4 252 975	6	284	172	456
mSI_462	CH4	1414	222	425	2	649
**mSI_466**	**CH4**	**4737**	**191**	**385**	**0**	**576**
**mSI_474**	**CH4**	**10 127**	**198**	**380**	**0**	**578**
**mSI_7**	**CH4**	**20 673**	**259**	**488**	**0**	**747**
**mSI_34**	**CH4**	**28 166**	**238**	**487**	**0**	**725**
**mSI_8**	**CH4**	**28 166**	**142**	**270**	**0**	**412**
**mTcCir312**	**CH4**	**32 259**	**277**	**516**	**0**	**793**
**mSI_35**	**CH4**	**33 618**	**183**	**348**	**0**	**531**
**mSI_2**	**CH4**	**43 494**	**191**	**335**	**0**	**526**
**mSI_542**	**CH4**	**63 388**	**33**	**68**	**0**	**101**
**mSI_303**	**CH4**	**119 995**	**299**	**578**	**0**	**877**
**mSI_458**	**CH4**	**136 890**	**208**	**399**	**0**	**607**
**mSI_460**	**CH4**	**139 590**	**198**	**406**	**0**	**604**
**mSI_308**	**CH4**	**139 780**	**211**	**408**	**0**	**619**
**mSI_309**	**CH4**	**141 679**	**205**	**411**	**0**	**616**
**mSI_310**	**CH4**	**142 517**	**193**	**383**	**0**	**576**
**mSI_315**	**CH4**	**233 706**	**301**	**563**	**0**	**864**
**mSI_402**	**CH4**	**246 098**	**259**	**506**	**0**	**765**
**mSI_535**	**CH4**	**252 815**	**110**	**232**	**0**	**342**
mSI_411	CH4	258 684	125	242	1	368
mS_413	CH4	270 916	118	253	1	372
mSI_39	CH4	278 179	277	421	1	699
mSI_42	CH4	343 424	109	233	2	345
mSI_46	CH4	428 250	119	230	2	352
mSI_54	CH4	751 986	55	127	4	521
mSI_294	CH4	1 686 245	109	248	16	373

Skewed segregations were observed in the chromosome 1 (CH1) and chromosome 4 (CH4) regions: 22 and 26 markers, respectively, defined in these regions, allowed the identification of restricted regions (in bold) with a complete absence of one of the three genotypes expected from the F_2_. The number of each genotype is indicated at the level of each marker: (a) homozygous for the ICS1 allele (Amelonado type); (h) heterozygous; and (b) homozygous for the SCA 6 allele.

In the skewed CH4 region, 26 markers segregating in the F_2_ progeny could be defined at the top of the chromosome (Table 1). The expected a/h/b genotype segregation for this F_2_ was 1/2/1, whereas only two genotypes, a and h, were observed at the top of CH4, within a region without recombinants delimitated by the markers mSI_462 (1414 bp), and mSI_411 (258 684 bp) (Table 1), and with a segregation of 1/2. Part of this F_2_ population had been tested for self-incompatibility in a previous study (Yamada *et al.*, 2010) and the QTL for self-compatibility, evaluated by the percentage of fruit set 14 d after self-pollination, was identified in the same region of CH4 as the skewed segregations, with the *Sf* Amelonado allele providing self-compatibility when homozygous.

Interestingly, the origin of the absent genotype is not the same on the two loci located on CH1 (genotype homozygous for Amelonado allele) and CH4 (genotype homozygous for SCA 6 allele).

### Skewed segregations observed in other progenies

Segregations were observed in several other progenies using three markers, mSI_103, mSI_107, and mSI_460, located in CH1 and CH4 regions, identified by fine mapping (Table 2).

**Table 2.** Segregations observed at the level of CH1 and CH4 loci for other progenies

Progeny	Marker	Chromosome	Parental genotypes	Number of seeds analysed	Expected genotypes	Number of seeds having the genotype
IMC60×IMC60 self-incompatible	mSI_460	CH4	133–145 × 133–145	39	**145**–**145**	**0**
					133–145	22
					133–133	17
	mSI_103	CH1	223–228 × 223–228	38	228–228	12
					223–228	19
					223–223	7
	mSI_107	CH1	233–245 × 233–245	38	233/233	12
					233/245	19
					245/245	7
BR36×BR36 self-compatible	mSI_460	CH4	161–161 × 161–161	34	161–161	34
	mSI_103	CH1	223–225 × 223–225	34	**223**–**223**	**0**
					223–225	20
					225–225	14
	mSI_107	CH1	241–245 × 241–245	33	**245**–**245**	**0**
					241–245	21
					241–241	12
BR59×BR59 self-compatible	mSI_460	CH4	161-161 × 161-161	109	161–161	109
	mSI_103	CH1	223–225 × 223–225	109	**223**–**223**	**0**
					223–225	77
					225–225	32
	mSI_107	CH1	241–245 × 241–245	108	**245**–**245**	**0**
					241–245	76
					241–241	32
UF676×ICS95 cross-compatible	mSI_460	CH4	134–161 × 134-134	87	134–134	43
					134–158	44
	mSI_103	CH1	223–228 × 223–228	87	223–223	26
					223–228	39
					228–228	22
	mSI_107	CH1	239–245 × 239–245	87	239–239	23
					239–245	38
					245–245	26
Reference genotypes						
Amelonado Mat1-6	mSI_460	CH4			161–161	
	mSI_103	CH1			223–223	
	mSI_107	CH1			245–245	
Criollo B97-61	mSI_460	CH4			134–134	
	mSI_103	CH1			228–228	
	mSI_107	CH1			239–239	
Scavina 6	mSI_460	CH4			140–149	
	mSI_103	CH1			225–225	
	mSI_107	CH1			241–243	

Segregations were observed for the loci in CH1 and CH4 for several progenies. The self-progeny obtained from the self-incompatible clone IMC60, using a pollen mentor, showed a total absence of one of the three expected genotypes, but only at the level of the CH4 locus. Two self-progenies were produced from BR36 and BR59, which are two self-compatible clones from the F_2_ progeny only heterozygous for the CH1 locus, with an Amelonado allele associated with a Scavina 6 allele: a total absence of the genotype homozygous for the Amelonado allele was observed for the locus of CH1. The last progeny (UF676×ICS95) was produced from a compatible cross between two Trinitario genotypes, both heterozygous for the CH1 locus, and associating an Amelonado allele with a Criollo allele: no skewed segregation was observed in this progeny, and all three expected genotypes were present. The complete absence of expected genotypes is highlighted in bold.

#### Self-progeny from IMC 60

A total of 39 self-fertilized individuals were obtained from 550 seedlings produced after pollinations made on IMC 60, another SI clone, using a mixture of compatible and incompatible pollen. Skewed segregations, with a complete absence of one homozygous genotype, were only observed in the CH4 region with mSI_460, while the three possible genotypes were all observed in the CH1 region with mSI_103, indicating the absence of gametic selection at the CH1 locus level.

#### Segregations observed in the SC F_2_ plants, homozygous for the CH4 loci (Sf/Sf) and heterozygous for the CH1 loci, after self-pollinations

Plants homozygous for the *Sf*/*Sf* CH4 locus are SC. In order to determine whether heterozygosity at the level of the CH1 locus could lead to skewed segregation even if associated with the *Sf*/*Sf* genotype at the CH4 locus, the F_2_ seedlings BR36 and BR59, homozygous *Sf*/*Sf* for the CH4 locus and heterozygous for the CH1 locus, were self-pollinated. For both the 109 seedlings from the selfing of BR59 and the 36 seedlings from BR36, and in common with the F_2_ progeny, there was a total absence of the ‘a’ genotype corresponding to plants homozygous for the Amelonado allele (mSI_103-223) for the CH1 locus (Table 2). These results show clearly that even if the plants are SC due to the *Sf*/*Sf* CH4 genotype, the CH1 locus is still functional, and a genotypic selection could still be observed. The existence of genotypes homozygous for the CH1 locus (as for the Amelonado variety) shows that the CH1 allele is not a lethal one, and instead it is firmly involved in the SI system and leads to genotype selection even if the plant is SC.

#### Segregations observed in another progeny involving the Amelonado CH1 allele

We analysed the segregations in the progeny of UF 676×ICS 95. Each Trinitario parent of this progeny, a hybrid between Amelonado and Criollo genotypes, is heterozygous for the mSI_103 (223) Amelonado allele and the mSI_103 (228) allele that originates from Criollo. No skewed segregation was observed in this progeny (Table 2).

A lack of fertilisation between both gametes containing the Amelonado allele (mSI_103-223) was observed when the SCA 6 allele (mSI_103-225) was associated with the Amelonado allele in the parents of the progeny (for example BR59×BR59 and BR36×BR36). This was not the case when the Amelonado allele was associated with the Criollo allele (mSI_103-228) in the parents of the progeny (as in UF 676×ICS 95). These observations probably reflect a dominance effect of the SCA 6 allele towards the Amelonado CH1 allele to generate the incompatibility reaction.

### Origin of genotypic selection in self-pollinated young fruits of BR59

In order to check whether the genotypic selection provided by the CH1 locus could result from gamete non-fusion, the proportion of fertilized ovules was assessed in young fruits of BR59 7 d after self-pollination. If the skewed segregations observed in the CH1 region reflect gamete non-fusion events, a proportion of 25% aborted ovules would be expected, corresponding to the theoretical proportion of ‘a’ genotypes eliminated after the self-fertilization of the heterozygous BR59 plant. Two types of hand-pollination were performed on BR59 trees: self-pollination and pollination using pollen from CCN 51, a SC clone taken as a control. A total of 316 ovules were observed after self-pollination and 217 after cross-pollination, and 11% and 9.2% of aborted ovules were observed, respectively. This similar proportion of aborted ovules, due to the intrinsic fertility of the clone, shows that the SI reaction controlled by the CH1 locus is expressed before the stage of gamete fusion, allowing the fertilization of all ovules in the ovary even in the case of the SI reaction. This is in contrast to the CH4 locus involved in fruit drop linked to gametic non-fusions in SI clones such as IMC 60 that has been observed by some authors.

### Association studies for fruit setting linked to self-incompatibility

**Fig. 1.**
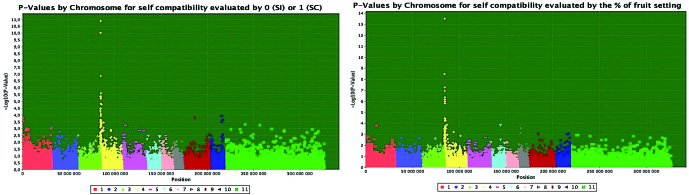
Genome-wide association study (GWAS) of self-incompatibility traits. GWAS-derived Manhattan plots showing significant *P*-values associated with self-incompatibility traits. Left: a qualitative variable was used with a score of 0 for self-incompatible trees (less than 10% of fruit setting after self-pollination) and 1 for self-compatible trees (more than 30% of fruit setting after self-pollination). Right: a quantitative variable: the percentage of fruit setting, assessed 14 d after self-pollination. A total of 16 480 genome-wide SNP markers identified by GBS were used for this analysis to generate the data, which was analysed using the MLM procedure of the Tassel software. Significant associations for CH1 to CH10, and for unmapped SNP (11) are shown by dots for a threshold of *P*-values <3e^–5^ [or −log_10_(*P*-value)>5.3], corresponding to a FDR cut-off *q*-value <0.05.

Using the Structure software, the number of sub-populations of the population of 570 individuals studied by GWAS was estimated as being *K*=9, and the contribution of individuals to the nine sub-groups was taken as a covariate in the GWAS analyses. Marker/incompatibility trait association studies using 16 480 SNPs were conducted on the 570 individuals coded with a 0/1 score for the SI/SC trait, and revealed positive associations for 11 SNP markers, all located at the top of CH4 from positions 189 447 bp to 1 328 172 bp (Table 3, Fig. 1). Only one marker, 3673000|F|0–37:G>A located at position 189 447 bp, was within the restricted region identified by fine mapping, which contained 36 other SNP markers subjected to GWAS. This marker is located inside the *Tc04_g000230* gene, an ortholog to the *GEX1* gene of *Arabidopsis thaliana*, and it had one of the two lowest values for *P* and *q* resulting from the statistical analysis, indicating that the candidate gene could be strongly linked to this marker. No association was detected in CH1. Similar results were obtained with the 388 individuals evaluated for SC/SI by the percentage of fruit setting, with eight positive associations located from positions 189 447 bp to 558 838 bp, and the same positive marker 3673000|F|0–37:G>A located in the region of fine mapping. These observations revealed that only the CH4 locus is associated with fruit drop, not the CH1 locus.

**Table 3.** Significant marker/self-incompatibility trait associations identified by GWAS

Marker	Chromosome	Position	*F*	*P*-value	*q*-value	Marker effect
SNP-SI/SC (0/1)						
3673000|F|0--37:G>A	CH4	189 447	24.16	9.17 e^–11^	7.56 e–7	0.087
3228529|F|0--41:C>G	CH4	274 083	10.56	3.16 e^–5^	4.73 e^–2^	0.038
3253696|F|0--14:T>C	CH4	283 464	12.55	4.69 e^–6^	1.19 e^–2^	0.044
3228956|F|0--38:G>A	CH4	291 211	12.67	4.16 e^–6^	1.19 e^–2^	0.045
3226653|F|0--38:G>T	CH4	325 666	26.24	1.29 e^–11^	2.13 e^–7^	0.093
3251567|F|0--25:T>C	CH4	427 186	16.24	1.4 e^–7^	7.69 e^–4^	0.057
3251143|F|0--28:A>G	CH4	461 808	12.24	6.29 e^–6^	1.30 e^–2^	0.043
3253828|F|0--22:T>G	CH4	515 636	12.47	5.06 e^–6^	1.19 e^–2^	0.044
3667093|F|0--48:T>C	CH4	558 838	13.19	2.53 e^–6^	1.04 e^–2^	0.046
3250663|F|0--32:G>C	CH4	581 276	11.36	1.46 e^–5^	2.67 e^–2^	0.040
3223093|F|0--65:T>A	CH4	1 328 172	11.18	1.73 e^–5^	2.85 e^–2^	0.039
SNP-SI/SC (% fr. set)						
3673000|F|0--37:G>A	CH4	189 447	20.65	3.22 e^–9^	2.65 e^–5^	0.109
3253696|F|0--14:T>C	CH4	283 464	17.53	5.28 e^–8^	2.90 e^–4^	0.092
3228956|F|0--38:G>A	CH4	291 211	16.75	1.07 e^–7^	4.41 e^–4^	0.087
3226653|F|0--38:G>T	CH4	325 666	33.83	3.12 e^–14^	5.14 e^–10^	0.177
3224400|F|0--35:A>G	CH4	432 831	15.22	4.4 e^–7^	1.45 e^–3^	0.079
3251143|F|0--28:A>G	CH4	461 808	14.70	7.15 e^–7^	1.96 e^–3^	0.077
3253828|F|0--22:T>G	CH4	515 636	14.33	1.01 e^–6^	2.38 e^–3^	0.075
3667093|F|0--48:T>C	CH4	558 838	13.84	1.58 e^–6^	3.25 e^–3^	0.072

A GWAS was carried out with the mixed linear model procedure implemented in Tassel 5.2.31 software for 570 individuals evaluated for self-incompatibility, with the trait coded as (0) for self-incompatible, SI, or by (1) for self-compatible, SC. Among them, 388 were also evaluated by the percentage of fruit setting after self-pollination. The population genotyping was carried out with 16 480 SNPs obtained by GBS. Significant associations were identified for a False Discovery Rate cut-off <0.05 after computing *q*-values from the *P*-values.

### Search for candidate genes potentially involved in the self-incompatibility system in the CH1 and CH4 genome regions and analysis of their expression

**Fig. 2.**
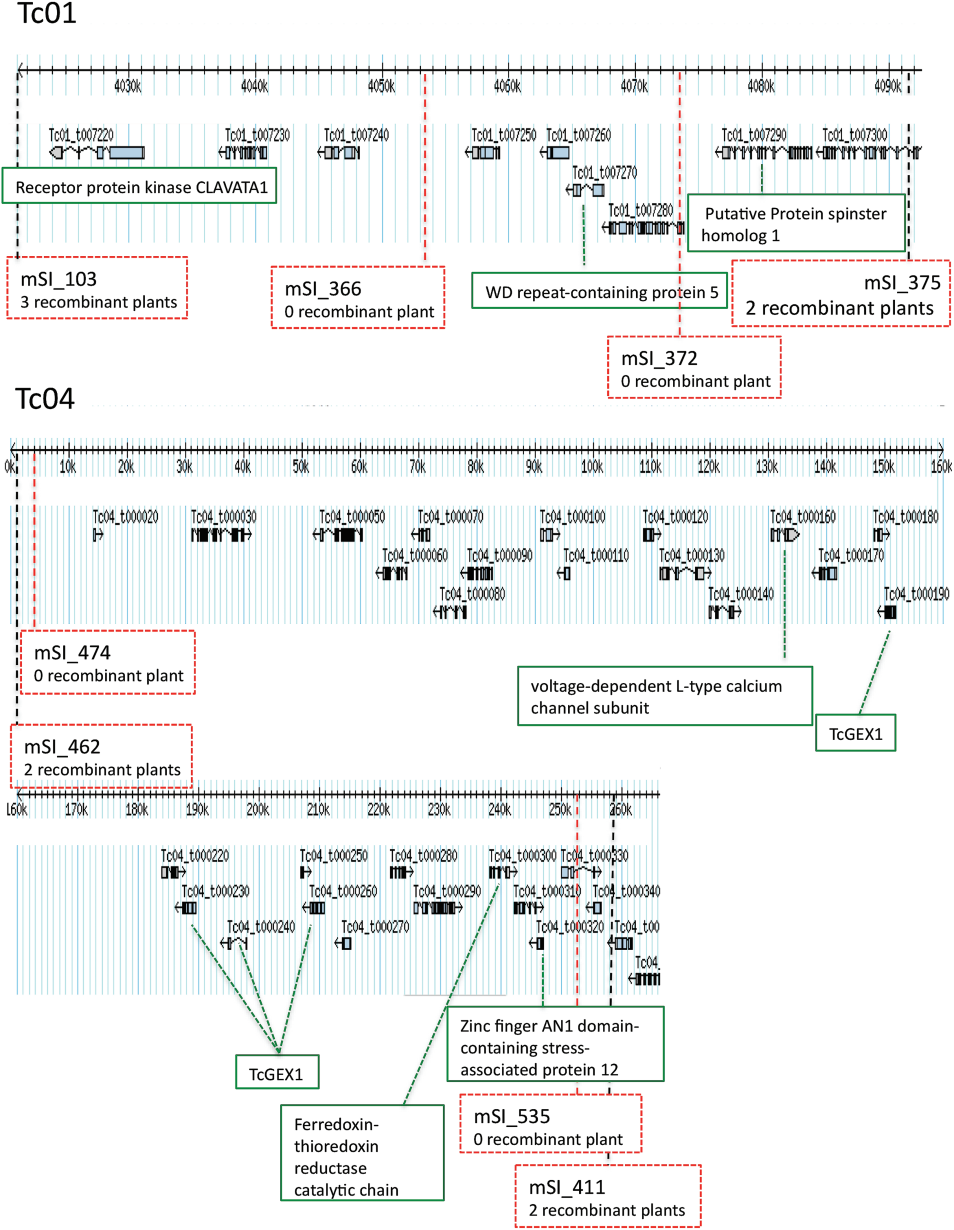
Schematic representation of CH1 and CH4 regions without recombinant plants. The SSR markers (mSI_x) delimiting the genomic regions of CH1 and CH4 without recombinant plants in the F_2_ progeny (with a total absence of one F_2_ genotype) are indicated in this diagram, as extracted from the cacao genome browser V1 (http://cocoa-genome-hub.southgreen.fr/gbrowse), as well as the candidate genes identified in these regions.

Searches for candidate genes potentially involved in cocoa self-incompatibility were first made in the CH1 and CH4 regions identified by fine mapping (Fig. 2), and the expression of 12 candidate genes was analysed during SC and SI reactions (see Supplementary Table S4).

#### CH1 region

No recombinant plants were identified within a region of 70.3 kb delimited by the markers mSI_103 and mSI_375, respectively located at the positions 4 021 267 bp and 4 091 577 bp of chromosome 1 (with a total absence of the ‘a’ genotype). In this region, only nine genes have been annotated in the whole-genome sequence V1 of Criollo (Argout *et al.*, 2011), from *Tc01_g007220* to *Tc01_g007300*. Among them three genes are considered as possible candidate genes, as follows.

**Fig. 3.**
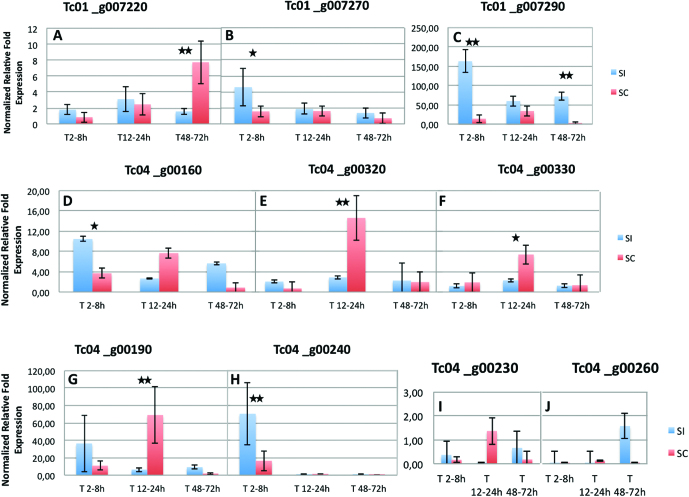
Relative expression of *T. cacao* CH1 and CH4 candidate genes differentially expressed during SI and SC reactions. The expression of 10 candidate genes was analysed by qPCR at several time points after SI and SC pollinations: 2–5–8 h, 12–24 h, and 48–72 h. Data are relative expression, in relation to unpollinated ovules, after normalization against two housekeeping genes. (A) Tc01_g0007220, orthologous to a leucine-rich repeat receptor-like serine/threonine-protein kinase BAM1; (B) Tc01_g0007270, orthologous to a transducin WD-40 repeat-containing protein; (C) Tc01_g007290, orthologous to a putative transmembrane transporter; (D) Tc01_g000160, orthologous to a voltage-dependent L-type calcium channel; (E) Tc04_g000320, orthologous to a zinc finger AN1 transcription factor; and (F) Tc04_g000330, orthologous to an ARM repeat-containing protein ARC1. (G–J) Orthologous to GEX1 genes: (G) Tc04_g000190, (H) Tc04_g000240, (I) Tc04_g000230, and (J) Tc04_g000260. Differential expression was tested by Fisher’s exact test: *P<0.05, **P<0.01.

*Tc01_g007220* is an ortholog of *A. thaliana* BAM1. BAM1 (*At5g65700*) and BAM2 (*At3g49670*) encode CLAVATA1-related leucine-rich repeat receptor-like kinases, which have an important role in early anther development (Hord *et al.*, 2006). DeYoung *et al.* (2006) have shown that BAM1 and BAM2 are involved in the development of both male and female gametophyte development. *Tc01_g007220* is differentially expressed between SC and SI reactions at later stages, with a higher expression during SC reactions (Fig. 3A).

*Tc01_g007270* is homologous to the COMPASS-like H3K4 histone methylase component WDR5a from *A. thaliana* (*At3g49660*). It is a transducin WD-40 repeat-containing protein acting as a site of protein–protein interactions and plays central roles in biological processes (Stirnimann *et al.*, 2010). It has also been reported that WD40 could be involved in the last step of the ubiquination cascade that leads to protein degradation, an important step in the self-incompatible reactions (Wang *et al.*, 2004).

*Tc01_g007290* encodes a putative transmembrane transporter, part of the major facilitator superfamily (MFS-1), and is homologous to the *A. thaliana At5g65687* gene, a probable sphingolipid transporter spinster homolog 1. According to the STRING network (http://string-db.org/), this gene is also predicted to interact with several genes involved in ubiquination. *Malus domestica* is characterized by a S-RNase-based GSI (Cheng *et al.*, 2006), and when S_Rnase is unable to enter in the pollen tube, due to the silencing of the transporter MdABCF (localized in the pollen tube membrane), this leads to a SC reaction.

*Tc01_g007270* and *Tc01_g007290* are differentially expressed between SC/SI reactions at early stages (2 to 8 h), with a much higher expression observed for *Tc01_g007290* during SI reactions (Fig. 3B–C). Observations at later stages (48–72 h) showed that *Tc01_g007270* expression levels decreased and became similar to those observed in unpollinated ovaries (control). In contrast *Tc01_g007290* expression remained at a high level and there was differential expression between the SI and SC reactions, with increased expression for SI reactions. *Tc01_g007290* could be involved in transport of molecules during the SI mechanism.

Immunolocalization experiments conducted with *Tc01_g007270* and *Tc01_g007290* showed that these genes are expressed in the cell layers of the style where pollen tubes are growing, and in cell layers surrounding the embryo sac.

**Fig. 4.**
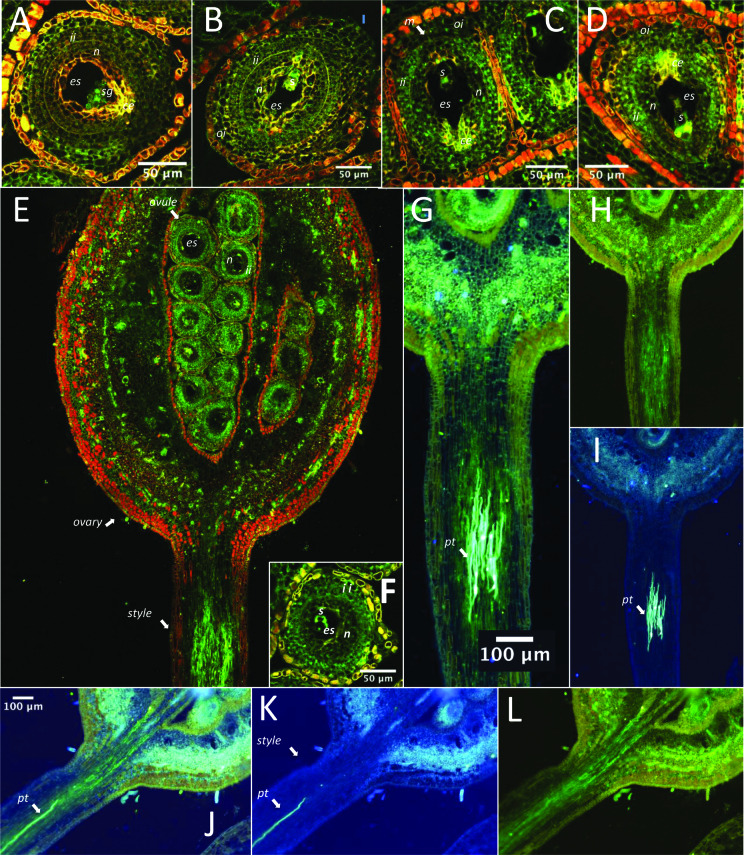
Immunolocalization of the Tc01_g007270 protein. (A–F) Confocal microscope images of sections of ovules, ovary, and style of the Scavina 6 accession either self-pollinated (self-incompatible, SI) or pollinated by a compatible pollen (SC) and probed with a Tc01_g0007270 antibody (labelled green). (A) Controlled hybridization labelled with pre-immuned rabbit serum for an ovule subjected to SC pollination. (B–F) Sections of ovules and ovary probed by a Tc01_g0007270 antibody: (B) unpollinated, (C) SC at 8 h, (D) SI at 8 h, (E) SC at 24 h, and (F) SI at 24 h. The Tc01_g0007270 protein appeared mostly present in the inner integument cells for the SC and SI reactions. (G–L) Images (Nikon microscope) of pollen tube germination at 8 h (J–L) and 24 h (G–I, same style as shown in image E) probed with Tc01_g0007270 antibody and stained with anilin blue. (I, K) Visualization of pollen tube germination (light blue), (H, L) Tc01_g0007270 protein immunolocalization, (G, J) merged images. The Tc01_g0007270 protein did not seem to be present in the pollen tubes themselves, as observed at 8 or 24 h after pollination, but seemed to be localized in the style tissues surrounding them. Abbreviations: es, embryo sac; s, synergid cell; sg, starch grains surrounding the polar nucleus; n, nucellus; oi, outer integuments; ii, inner integuments; m, micropyle; ce, chalazal end; pt, pollen tube.

*Tc01_g007270* was mostly expressed in the inner-integument cells and also in the nucellus during SC and SI reactions. Its expression can be also detected, but to a lower extent, in the same cell layers of unpollinated ovules. Its expression was not observed in pollen tubes at 8 or 24 h after SC pollination of SCA 6, but it could be observed in the style cells where pollen tubes were growing (Fig. 4).

**Fig. 5.**
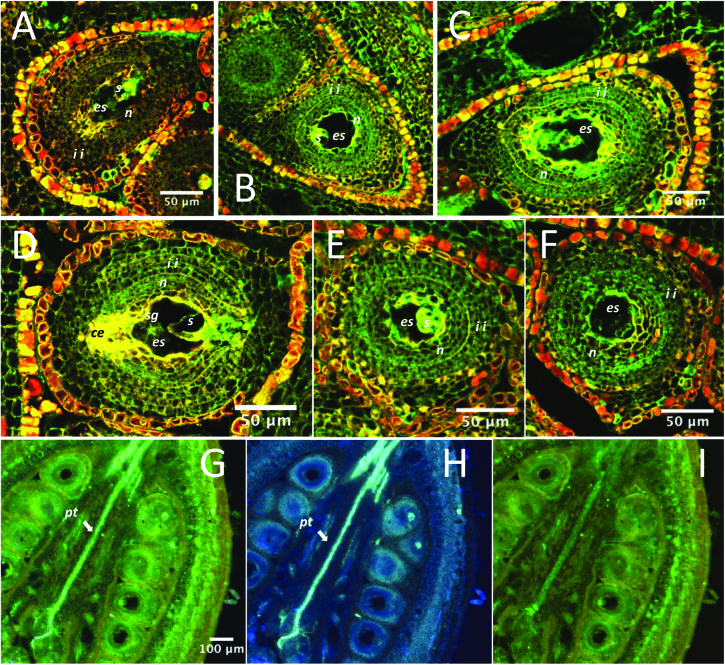
Immunolocalization of the Tc01_g007290 protein. (A–F) Confocal microscope images of sections of ovules of the Scavina 6 accession either self-pollinated (self-incompatible, SI) or pollinated by a compatible pollen (SC) and probed with a Tc01_g0007270 antibody (labelled green). (A) Controlled hybridization with pre-immuned rabbit serum for an ovule subjected to SC pollination. (B–F) Sections of ovules probed by a Tc01_g0007290 antibody: (B) SC at 8 h, (C) SI at 8 h, (D) unpollinated, (E) SC at 24 h, and (F) SI at 24 h. The Tc01_g0007290 protein appeared present in the nucellus and inner integument cells for SC and SI reactions. Its presence seemed to be reinforced in the cell layer separating the nucellus from the inner integument. (G–I) The presence of the Tc01_g0007290 protein was visualized as described in Fig. 4. The protein was not apparent in the pollen tubes. Abbreviations: es, embryo sac; s, synergid cell; sg, starch grains surrounding the polar nucleus; n, nucellus; ii, inner integuments; ce, chalazal end; pt, pollen tube.

*Tc01_g007290* was expressed in the nucellus and in the inner-integument cells, with a stronger expression observed in the cell layer separating the two. No difference was visible between SC and SI reactions. Its presence in pollen tubes was not observed at 8 or 24 h after SC pollination, or at 24 h after SI pollination, but it was observed in the style cells where pollen tubes were growing (Fig. 5).

#### CH4 region

The region of 257.270 kb identified by fine mapping includes 30 genes annotated in the Criollo genome V1. Among them, nine genes were identified as candidates potentially involved in the SI reactions, as follows.

*Tc04_g000160* is homologous to the voltage-dependent L-type calcium channel subunit alpha-1F gene from *Gossypium arboreum*. This gene could play a role in Ca^2+^ influx pathways, as has been shown in humans (Kotturi *et al.*, 2003) and in plants. It was demonstrated that Ca^2+^ influx into stigma papilla cells mediates SI signalling in the Brassicaceae, and it could be a common event in other types of SI systems. This gene is up-regulated during incompatible reactions (Iwano *et al.*, 2015). *Tc04_g000160* is differentially expressed between SC and SI reactions at early stages (2–8 h). Its higher expression during SI reactions is in agreement with the Ca^2+^ pathway described in the Brassicaceae during SI reactions (Fig. 3D).

*Tc04_g000170* is an ortholog to a KANADI transcription factor, potentially interacting with an auxin factor response 3. It is not differentially expressed between SC/SI reactions.

*Tc04_g000190*, *Tc04_g000230*, *Tc04_g000240* and *Tc04_g000260* are homologous to *A. thaliana GEX1* (GAMETE EXPRESSED PROTEIN, *At5g55490*) genes potentially involved in early embryo development. They have a dual function during male and female gametophyte development and early embryogenesis, and are required for correct pollen maturation. In *A. thaliana*, *GEX1* is expressed in several tissues and several steps of embryogenesis: in the embryo sac before cellularization, the egg cell after cellularization, the zygote/embryo immediately after fertilization, and the male gametophyte, in both the vegetative cell and in the sperm cells (Alandete-Saez *et al.*, 2011). Using the STRING network, it was shown that in *A. thaliana*, *GEX1* interacts with the *HAPLESS2* gene involved in male fertility, and is essential for pollen tube guidance, successful gamete attachment, and fertilization.

Among *Tc04_g000190*, *Tc04_g000230*, *Tc04_g000240* and *Tc04_g000260*, *Tc04_g000230* and *Tc04_g000260* had a level of expression similar to those of unpollinated ovules, while the two others displayed a differential expression between SC/SI reactions compared to unpollinated ovules (Fig. 3G–J). *Tc04_g000240* showed a higher expression level, but only at early stages of the SI reaction. *Tc04_g000190* was significantly more expressed during the SC reaction during the period 12–24 h.

*Tc04_g000300* is ortholog to a ferredoxin thioredoxin reductase, and was not differentially expressed between SC/SI reactions.

*Tc04_g000320* is an ortholog of a zinc finger AN1 domain-containing stress-associated protein 12 (PMZ) gene from *A. thaliana*. According to the STRING network, PMZ interacts with several genes associated with protein degradation through the ubiquination complex. PMZ also interacts with *AtCDC48B*, a gene involved in cell division and growth processes that interacts with SNAREs, transmembrane proteins that are mainly responsible for membrane fusion and exchanges in the cell (Martens and McMahon, 2008). *Tc04_g000320* was significantly more expressed at the stage 12–24 h during the SC reaction, and also constitutes a good candidate gene potentially involved in variations in self-incompatibility (Fig. 3E).

*Tc04_g000330* is an ortholog of the ARM repeat-containing protein ARC1 gene from *Brassica napa* (*AGP76183.1*). The ARC1 protein is involved in SI signalling in *Brassica* and targets proteins for degradation during the SI response, promoting the ubiquitination and proteasomal degradation compatibility factors in the pistil (Stone *et al.*, 2003). In our experiments, *Tc04_g000330* was differentially expressed at the stage 12–24 h, where a higher expression was observed for the SC reaction compared to unpollinated ovaries or the SI reaction, which is not in agreement with what is expected during the SI reaction (Fig. 3F).

### Self-compatibility/incompatibility predictions

Using SSR markers located in the CH1 and CH4 regions, we genotyped a population of 710 individuals assessed for self-incompatibility in order to test for genotypes or genotype combinations that are able to predict SC or SI plants.

#### Genetic diversity and allele specificity

Two old *T. cacao* varieties, Amelonado and Criollo, are the ancestors and the main sources of self-compatibility alleles in the species. Both are completely homozygous, and are the origin of most of varieties under cultivation worldwide. Specific Amelonado and Criollo alleles were identified among the CH4 markers, predicting SC trees homozygous for these alleles.

The 161-bp allele of mSI_460 is specific to Amelonado. The presence of this allele with a low frequency in the Nanay and Nacional groups probably reflects introgression of Amelonado in some individuals of these two groups. Another allele of mSI_460, allele 149, is only shared by the SC Amelonado genotypes (mainly present in African Amelonado genotypes), and the Contamana SI genotypes (particularly SCA 6, a high-performance progenitor, widely used in breeding programs; see Supplementary Table S5). The combined use of markers mSI_303 and mSI_460 enables discrimination between the two haplotype origins: the mSI_460-149 allele is shared by the Amelonado and Contamana genotypes, whereas the mSI_303-227 allele is present in Amelonado genotypes but absent in the Contamana group, and the mSI_303-229 allele can be present among Amelonado genotypes but is always absent in Contamana group.

The mSI_460-159 allele is shared by some Amelonado and Purus genotypes; however, the mSI_303-229 allele may allow discrimination between Amelonado and Purus genotypes in some cases.

The allele 217 of mSI_303 is specific to Criollo and could be used to screen for SC plants.

#### Genotype analysis

Allele combinations other than those identified in Amelonado and Criollo could result in SC plants. Dominance relationships exist between S-alleles and the multi-allelic status of SSR markers allows a better discrimination of S-haplotype interactions.

When only one genotype was considered, 24 genotypes from seven markers were significantly associated with cocoa self-incompatibility by Fisher’s Exact test (*q*-value<0.05). Among those genotypes, when present, 16 resulted in a higher frequency of SC plants and 10 resulted in a higher frequency of SI plants (Supplementary Table S6). Among those associated with SI, when present in a given individual, the genotypes 231/231 and 231/233 (locus mSI_303), 194/197 (mSI_7), and 155/161 (mSI_460), resulted in a chance of at least 95.6% of being SI (Supplementary Table S6). Trees with the six other genotypes resulted in a chance of at least 74.8% of being SI. Among the 16 genotypes associated with a higher number of SC plants, the genotypes 161/161 (mSI_460), 225/227 (mSI_303), 292/318 (mSI_458), and 132/149 (mSI_460) presented at least a 91.7% chance of being SC, with a very low chance (<8.3%) of being SI. The allele 161 of mSI_460 is specific to the SC Amelonado variety, which is homozygous for this allele, and 100% of individuals homozygous for this allele are SC. When two genotypes were considered, 178 combinations of genotypes were significantly associated with SC/SI status in cacao, with 134 combinations resulting in more SC clones and 44 in more SI clones. The addition of one more marker in some cases improved the discrimination of SC/SI individuals. For some specific marker combinations, the SI/SC ratio of trees was lower than 1/56; the discrimination of SI was also improved with some other genotype combinations (Supplementary Table S6). Predictions of SC/SI status obtained from a combination of three genotypes were not significantly more efficient than predictions based on one or two genotypes.

The use of any of the 26 genotypes listed in Supplementary Table S6 will result in an increased frequency of SC plants in the population. However, the loci mSI_460 and mSI_303 with 22 and 19 alleles, respectively, appear as the best predictors of SC/SI status. Combining both markers could greatly increase the efficiency of early selection of SC or SI genotypes.

#### Allele effect

Among the 128 alleles tested, 43 were significantly associated with SC/SI status by the Fisher’s Exact test (Supplementary Table S7). It can be seen that the probability of a plant being SC is rarely higher than 50%. In contrast, the presence of one or two copies of several alleles seems to confer SI to cocoa plants. This is the case for the allele 225 of mSI_103, the presence of which in the F_2_ progeny and in BR36 and BR59, when associated with mSI_103-223, prevents the formation of a homozygous plant for the other allele (mSI_103-223). In the population studied for predictions, the probability of an individual having two copies of this allele being SI was 90%. Fifteen alleles from different markers give plants a probability of being SI that is greater than 80%. The allele effects could reflect the relationships of dominance that exist between incompatibility alleles: the presence of particular SSR alleles, such as mSI_7/197 or mSI_460/155, are probably linked to some dominant incompatibility alleles, giving the plants a low probability (0.07) of being SC.

## Discussion

*Theobroma cacao* has a complex incompatibility system under the control of several genes. It has already been described as a late incompatibility system having both gametophytic and sporophytic aspects and dominance relationships between alleles (Cope, 1958, 1962; Glendinning, 1960). Our results confirmed its gametophytic and sporophytic features and led to the identification of two independent loci involved in the SI system through two different mechanisms. Indeed, the CH1 locus, identified for the first time in this study, does not function as a LSI system expressed at the stage of gametic non-fusions, nor as classical GSI or SSI systems. The CH1 locus has traits of both gametophytic and sporophytic systems: it depends upon the nature of the alleles present, as for a GSI (for example, when selfing BR36 or BR59, only the homozygous genotype mSI_103-223 was eliminated, in contrast to the homozygous genotype mSI_103-225 which could be observed in the progenies), but it depends also upon specific interactions between them, as in a SSI (for example, the mSI_103-223 allele generates a SI reaction only when associated with the mSI_103-225 allele and not when associated with itself, as in the SC Amelonado variety, or when associated with the mSI_103-228 allele, as in ICS95).

The proteins of candidate genes of the CH1 region (*Tc01_g0007270* and *Tc01_g0007290*) did not seem to be present in the pollen tubes observed at 8 or 24 h after pollination, but were apparent in the style cells where there were growing pollen tubes. The expression of these genes in the female tissues could result from interactions between male and female tissues that depend upon the specific allele combination and parental allele interactions, and result in the prevention of fertilization between some gametes. This could explain the CH1 locus segregation observed in BR36 and BR59 after self-pollination, with 2/3 heterozygous plants and 1/3 homozygous plants. Moreover, in these cases all ovules were fertilized which reflects a gametic selection that happened prior to the gamete fusion step. This selection could involve a lack of penetration or migration of the sperm nuclei in the embryo sac as was already observed in SCA 24 (closely related to SCA 6) (Ford and Wilkinson, 2012).

A region of 70.3 kb without recombinants plants, identified by fine mapping, contained only nine genes. Three of them were differentially expressed: *Tc01_g007220* is ortholog to a gene involved in the development of the gametophyte, and *Tc01_g007270* and *Tc01_g007290* are orthologous to genes known to be involved in GSI in other species and involved in protein–protein interactions and protein degradation, respectively, and also in transport of S-RNAse in the pollen tube. Through qPCR analysis, it was determined that *Tc01_g007270* and *Tc01_g007290* are up-regulated in the earlier stages of SI reactions (2 to 8 h) compared to SC reactions, which is in accordance with their potential interactive role with putative gametophytic S-locus genes not present in this locus. In *Solanum chacoense*, a single dominant gene (*Sli*) with sporophytic action that inhibits gametophytic S gene expression in the pollen has been identified (Hosaka and Hanneman, 1998). The candidate genes identified for the CH1 locus could have a similar role as modifier genes, in interaction with gametophytic genes located in other genome regions.

The CH4 locus is involved in the SI reaction through a different mechanism, strongly associated with fruit drop. All positive associations identified by the GWAS were determined in the CH4 region where a gametic selection (probably linked to the observations on gamete non-fusions made by several authors: Cheesman 1927; Cope 1939*b*, 1940, 1958, 1962; Posnette, 1940; Knight and Rogers, 1955; Bouharmont, 1960; Ford and Wilkinson, 2012) was also observed in the F_2_ progeny under study, as in the self-progeny of IMC 60, another SI clone. In these two progenies, only the use of mentor pollen, which prevented the fruit from dropping, had allowed the seed development in ovules where gametic fusions were effective. A region of 257.270 kb, without recombination and containing 30 genes was also identified in CH4. A GWAS conducted on a large population of 570 individuals identified 11 SNP markers significantly associated with fruit setting in the top of CH4. However, only one SNP marker (3673000|F|0–37:G>A), located in *Tc04_g000230*, an ortholog to the *GEX1* gene from *A. thaliana*, was significantly associated with fruit setting in the CH4 region identified by fine mapping and thus probably close to the gene controlling the CH4 locus. Among the other three closely linked *TcGEX1* orthologous genes (*Tc04_g000190*, *Tc04_g000240*, and *Tc04_g000260*), *Tc04_g000190* was the most expressed in SC reactions in comparison with unpollinated ovules, at the stage 12–24 h. The *GEX1* genes, involved in male and female gametophyte development and in early embryo development in *A. thaliana*, could be involved in the late incompatibility reaction of *T. cacao,* acting at the ovule level and controlled by the CH4 locus. Recently, it has been observed that the non-fusion of gametes, as observed after pollination of *T. cacao* using incompatible pollen, could result from two different and independant mechanisms: an incomplete migration of the sperm nucleus, which fails to reach the female nuclei, or a successful sperm nucleus migration and reaching of the female nuclei, but followed by a non-fusion of gametes (Ford and Wilkinson, 2012). The role of *GEX1* genes, interacting with the *HAPLESS2* gene that is known to be essential for pollen tube guidance, successful gamete attachment and fertilization (von Besser *et al.*, 2006; Mori *et al.*, 2006), could be determinant in this late manifestation of SI in *T. cacao*. Until now, few results have been reported on molecular mechanisms potentially involved in LSI (Zhou and Zheng, 2015) and the *GEX1* genes constitute good candidates for its control. However, *Tc04_g000160*, *Tc04_g000320*, and *Tc04_g000330*, orthologs to genes involved in SI signalling in *Brassica* and protein degradation during the SI response, cannot be ruled out and could also participate in the pathway leading to self-incompatibility.

The practical output from this study is the identification of markers that could help breeders to select for SC plants in cocoa populations at an early stage in the breeding process. Indeed, the identification of highly polymorphic SSR markers in the CH4 region identified by fine mapping gives the potential to have a strong linkage disequilibrium between SSR alleles and incompatibility alleles, leading to a good ability of SSR to predict SI or SC genotypes. Specific Amelonado and Criollo alleles, identified in this study, will be particularly efficient in predicting SC varieties.

*T. cacao* has developed an efficient system to maintain genetic variability, based on two different and independant mechanisms of gamete selection. However, beyond the progress already achieved in deciphering the *T. cacao* SI system, made possible by advances in the fields of genomic technology and whole-genome sequencing, many questions still remain about its mechanism, which involves several loci for which no clear orthology with classical GSI or SSI S-locus receptors was found. Further investigations will be needed to study their potential interactions with other loci of the whole genome and to establish the links between incompatibility alleles and structural variations of candidate genes. This increased knowledge will help to achieve better identification of S-alleles and consequently will allow for better control of selection for the self-compatibility trait in *T. cacao*.

## Supplementary data

Supplementary data are available at *JXB* online.

Table S1. Primer pairs defined in CH1 and CH4 regions.

Table S2. Primer pairs defined in candidate genes differentially expressed.

Table S3. Reference SSR profiles for a collection of genetic resources.

Table S4. Relative expression of candidate genes.

Table S5. Allelic frequencies of SSR markers located in CH1 and CH4 regions in the *T. cacao* genetic groups and populations studied.

Table S6. Significant predictions of self-compatibility/incompatibility based on genotype presence.

Table S7. Significant predictions of self-compatibility/incompatibility based on allele presence.

## Supplementary Material

Supplementary Tables S1-S7

Supplementary File
