# Supplementary material for: Deciphering the Theobroma cacao self-incompatibility system: from genomics to diagnostic markers for self-compatibility
Source: J Exp Bot. 2017 Oct 7;68(17):4775–90. doi: 10.1093/jxb/erx293 (PMC5853246; doi:10.1093/jxb/erx293)
Supplement: Supplementary Tables S1-S7 [file erx293_suppl_supplementary_tables_s1-s7.pdf]

# Supplementary file

### Table S1. Primer pairs defined in CH1 and CH4 regions.

| Markers | Chromosome | Position | type | 5'-3' forward primer | Tm | 5'-3' reverse primer | Tm | PCR product size (bp) |
|---|---|---|---|---|---|---|---|---|
| mSI_26 | CH1 | 3377732 | (AT)10 | TGCTTTCAGGCCAATACA | 56,2 | CCTCTTGCTTCCGGTTT | 56,3 | 145 |
| mSI_88 | CH1 | 3499444 | (TC)9(AC)9 | CGCCCTCCCCTTATTT | 55,9 | TCCTTTCCTTGCAGGTTT | 55,8 | 238 |
| mSI_89 | CH1 | 3525756 | (AT)9 | TTTTCCTTGTTCCTGCCT | 55,8 | ATTGAATGGGGTTGGTGT | 55,9 | 170 |
| mSI_32 | CH1 | 3649333 | (CT)8 | TGGGATGAATGAGAATGGT | 56,1 | TCATAACAACAACGGATTGAG | 56,2 | 203 |
| mTcCIR15 | CH1 | 3711664 | (TC)19 | CAGCCGCCTCTTGTTAG | 52,5 | TATTTGGGATTCTTGATG | 45,3 | 254 |
| mSI_73 | CH1 | 3790637 | (AT)14 | TTTACGGCTGCTTGTCC | 55,7 | AAGGTGCCCATAACTCAAA | 55,7 | 278 |
| mSI_101 | CH1 | 3935902 | (TC)12 | GATTGGCACGTCATCAAC | 55,7 | ATGCAATGAGAGGAGGAAA | 55,7 | 234 |
| mSI_102 | CH1 | 3966163 | (GA)7 | CAGTTTCCACTTCTAATCTTGT | 53,0 | TGTACTTTCCTATGTTTTACCC | 52,9 | 115 |
| mSI_140 | CH1 | 3988656 | (T)13 | CTCTGCGGTGGAAGAAA | 55,7 | AGTCATTGCCGACCTCTT | 55,6 | 229 |
| mSI_141 | CH1 | 4010921 | (T)13 | AACCTTGAAGCCAAGCA | 55,2 | GCATGTAGGGTAAAGCCA | 54,6 | 261 |
| mSI_103 | CH1 | 4021267 | (TG)6(TA)7 | CAGGCTGCCATTTTCTC | 55,1 | TCAAGGACTGCTCCAAAA | 55,2 | 209 |
| mSI_366 | CH1 | 4053385 | (T)13 | TCATCACACTCGAAAACCTC | 56,2 | ATCTTCTGACTTTTCAAACCCT | 56,2 | 236 |
| mSI_367 | CH1 | 4054418 | (T)14 | TTGTGCAATCGAGAAGG | 53,8 | ATCCACGTCGTCAAACA | 53,8 | 263 |
| mSI_369 | CH1 | 4057532 | (A)13 | CGATGCAGAAACTGATGAA | 55,7 | TGTCTTGCCTTTTGGGA | 56,0 | 278 |
| mSI_440 | CH1 | 4066036 | INDEL | GTCACTCCCATTTTCAAGTCTATTC | 57,9 | AACGAGGAATGATGGCAAGGT | 60,0 | 312 |
| mSI_370 | CH1 | 4070474 | (A)16 | CCTCAACCAAAACCCATC | 56,2 | TTTTGCCTGTTTTCTTGCT | 56,2 | 232 |
| mSI_372 | CH1 | 4073585 | (A)13 | GCGCTCTCTCGCAGTT | 56,1 | AATTTACTCGGCGCATTT | 55,9 | 140 |
| mSI_375 | CH1 | 4091577 | (A)12 | GTTGAAAGTTAGTCATTCCAGAG | 54,9 | TTGTGGAGACCAAGTGAAA | 55,0 | 257 |
| mSI_107 | CH1 | 4130575 | (AT)10 | GAAAATACTCCGTAACAACCA | 54,4 | ACCTTACCAACACCACACA | 54,6 | 221 |
| mTcCIR356 | CH1 | 4149062 | (TC)6 | GGGGTTGCTTTCTGCTT | 51,7 | AAAACTTGGCGGAGGAG | 52,8 | 163 |
| mSI_112 | CH1 | 4233257 | (TTGG)5 | GCTTGCACTGCTATCCCT | 56,5 | CGCCACTTACAAACCCA | 56,4 | 269 |
| mSI_113 | CH1 | 4252975 | (TA)12 | TTTCAGGATGTTAGATGGGA | 55,565 | ATGTCATCATCCAAGGCA | 55,58 | 258 |
| mSI_462 | CH4 | 1414 | INDEL | TTTAGGGTTTTTTAAAGGATGT | 51,8 | ATGCTTAAAAGTTTATGAATAAAACC | 53,7 | 198 |
| mSI_466 | CH4 | 4737 | INDEL | AAGGGCATTCTAAGCATTTTG | 55,1 | AATCCAAAAACTCCATAAAAACCA | 55,7 | 227 |
| mSI_474 | CH4 | 10127 | INDEL | AGGATGTTTAAGGTCATTCTAAGC | 56,8 | GGAGCTTCCAGTTTGTTTTCAC | 58,3 | 350 |
| mSI_7 | CH4 | 20673 | (AGA)8 | TTTCATGGAGGTTGGGA | 55,5 | GTTGCACAAAGGATGGG | 55,7 | 183 |
| mSI_34 | CH4 | 28166 | (AG)7 | CCCAACCAACCAATATCC | 56,0 | GAGGGATTCGACAGAGACA | 56,1 | 267 |
| mSI_8 | CH4 | 28166 | (CT)6...(TC)6 | GAGGGATTCGACAGAGACA | 56,1 | CCCAACCAACCAATATCC | 56,0 | 264 |
| mTcCir312 | CH4 | 32259 | (TC)6 | GCAACGCAACATTCTCC | 51,8 | ACGCAAAACCAAAACACA | 52,5 | 143 |
| mSI_35 | CH4 | 33618 | (AG)14 | TCCCGATAGCCTCAACA | 56,0 | ACAAATTCCTTCATCCCCT | 55,9 | 122 |
| mSI_2 | CH4 | 43494 | (TA)9 | CATCGAAAGTCAAGAAAAGG | 55,1 | ATTGAAATGGTGGTTTGGT | 55,1 | 268 |
| mSI_542 | CH4 | 63388 | INDEL | CTTTCAGGAGAATCCAAGCCA | 57,9 | TCAGCTCGCCTCATTTTGTT | 58,1 | 205 |
| mSI_303 | CH4 | 119995 | (AT)11 | CAAGTCGTTGGGAGGG | 55,7 | AAAGTTTCAATCCCATTTCC | 55,6 | 255 |
| mSI_458 | CH4 | 136890 | (TA)11 | GACACGAGATGTATCCTGACCA | 59,3 | TGCAACCGTGAGCATTTTGT | 59,3 | 284 |
| mSI_460 | CH4 | 139590 | (TC)8 | TGAGAACAAAGCCAAAGAAAGGA | 58,7 | CCGAGACAAAGCCCAGAAG | 58,2 | 117 |
| mSI_308 | CH4 | 139780 | (TC)10 | GCTTCTGGGCTTTGTCTC | 56,0 | CAGCACGGTAGCCATAGA | 55,8 | 279 |
| mSI_309 | CH4 | 141679 | (GA)13..(GA)9 | GGCTTGGAGGAGCAGA | 55,6 | GAGAAACAGAGAGCGACAGA | 55,8 | 260 |
| mSI_310 | CH4 | 142517 | (TA)6 | GGCCTTCATGGGTTTTAG | 55,6 | TTCAAATCCCCATTTTCC | 55,7 | 252 |
| mSI_315 | CH4 | 233706 | (TC)6 | CAAGGGGTCTTGGGTTT | 55,7 | AATGATGGCGATGGAGA | 55,7 | 206 |
| mSI_408 | CH4 | 236686 | INDEL | TGCAGAGGCCATGCGAGTAT | 61,7 | TGCACTTGAAAGAGGGGGAA | 59,2 | 244 |
| mSI_402 | CH4 | 246098 | INDEL | GCACATTCCCTTGCTGCT | 58,3 | CGTCATCTCCAACTACGCCA | 59,8 | 140 |
| mSI_535 | CH4 | 252815 | INDEL | AGTTGACTATCTTGCAAACCAATAC | 57,9 | GTTCGGCTAAGCAATGCACG | 60,5 | 313 |
| mSI_411 | CH4 | 258684 | INDEL | CGCCAGGCATTCTTACTCTT | 58,0 | ATACTGGACATCTTGTTGAATGAC | 57,0 | 274 |
| mS_413 | CH4 | 270916 | INDEL | TAAATGGTTGGGCTTTGGGGT | 60,1 | ACGCTTACCCTATTTTCACGATTCT | 60,6 | 117 |
| mSI_39 | CH4 | 278179 | (AT)15 | CCATTTATTGACACTGGGG | 56,2 | CTCTGCCCATGCTTCAC | 56,0 | 239 |
| mSI_42 | CH4 | 343424 | (AT)9 | AGCCTTCCAATGTGGTG | 55,2 | CAGTTGGGCTGGTGTCT | 55,3 | 209 |
| mSI_46 | CH4 | 428250 | (AT)8 | CTGCCACAGAATCACTCAA | 55,6 | AAATCCCACCACCTTCC | 55,5 | 258 |
| mSI_54 | CH4 | 751986 | (AC)8 | GCCAATGGACTCCTCAA | 55,2 | ACTGCTTTCCCACTCTCC | 55,1 | 257 |
| mSI_294 | CH4 | 1686245 | (GA)9...(AC)7 | ACAATGAGGTGAGGGGA | 54,3 | ATGTGAGATGTGGTGCCT | 54,5 | 238 |

Primers pairs were designed in the CH1 and CH4 regions displaying skewed segregations, in order to identify polymorphic SSR or INDEL (insertion/deletion) markers that can be used for fine mapping of these genome regions. Tm : primer melting temperature.

**Table S2. Primer pairs defined in candidate genes differentially expressed.**

| Gene | Putative function | Primer sequences | | Primer efficiency |
|---|---|---|---|---|
| | | 5'-3' Forward primer | 5'-3' Reverse primer | |
| **Tc04_g000050** | Isocitrate dehydrogenase (housekeeping gene) | GTTTCAATGATGTGAGATCATTATCTC | GGTTCAGATATGCAAGGGTTG | 1,75 |
| **Tc08_g003640** | Tubulin beta-6 chain (housekeeping gene) | ATTCCCCCGTCTTCACTTCT | TCTGCTCATCAACCTCTTTGG | 1,78 |
| **Tc01_g007220** | CLAVATA1-related Leucine rich repeat receptor like kinase | CCAACAATGAGGGAAGTGGT | GCTGGTCTTTCTGGTCCTTTG | 2 |
| **Tc01_g007270** | transducin WD repeat-containing protein 5 | GAGGGTCATAGTGACGCTGTT | CATGATCTCAAAGGGGTGCT | 2 |
| **Tc01_g007290** | Putative Protein spinster homolog 1 | AGCCATTGCTTGATGGAAAT | TCACGTATCCCCGTCCTTTA | 1,7 |
| **Tc04_g000160** | Voltage-dependent L-type calcium channel subunit alpha-1F | GACTGGATCGGCTCTTTCAG | TTCTGGAACCGCTGGTAATC | 1,62 |
| **Tc04_g000190** | Putative Protein *GEX1* | TTGTTGGCAGGACTATGTGC | CAACGCTTTGTTGCTTTGAA | 2 |
| **Tc04_g000230** | Putative Protein *GEX1* | GGTTCTTCGCTTGACAACAA | TTGTGTTGGAAAAGGTTGTGA | 2 |
| **Tc04_g000240** | Putative Protein *GEX1* | TTCTGGAGGATCCCCTTTTT | ATGCACTTGCACCAAAGTCA | 2 |
| **Tc04_g000260** | Putative Protein *GEX1* | TGTGGATTGGTCTTCTTGGA | TCTGGCATCAATTTGCCATT | 1,6 |
| **Tc04_g000320** | Zinc finger *AN1* domain-containing stress-associated | CGGGGATCCTAAATTCCG | CCGATTTCAGGCATTCGT | 1,7 |
| **Tc04_g000330** | U-box domain-containing protein 40-*ARC1* | GAGCTGGTGAAGGTGGAGAG | CACATGACCCGTTCTTTCCT | 1,73 |

Primers pairs designed in the 10 candidate genes differentially expressed between SC and SI reactions, located in the CH1 and CH4 genome regions, and in two housekeeping genes.

## Table S3. References SSR profiles in a collection a genetic resources.

| | Chromosome | CH4 | CH4 | CH4 | CH4 | CH4 | CH4 | CH4 | CH4 | CH4 | CH1 | CH1 |
|---|---|---|---|---|---|---|---|---|---|---|---|---|
| | mk position | 20673 | 33618 | 43494 | 119995 | 136890 | 139590 | 233706 | 236686 | 258684 | 4024677 | 4130575 |
| **Genetic group** | **marker / clone** | **mSi7** | **mSI_35** | **mSi_2** | **mSI_303** | **mSI_458** | **mSI_460** | **mSi_315** | **mSi_408** | **mSi_411** | **mSI_103** | **mSI_107** |
| **Amelonado** | **CATONGO** | 200/200 | 119/119 | 283/283 | 227/227 | 292/292 | 161/161 | 247/247 | 272/272 | 286/286 | 223/223 | 245/245 |
| **Amelonado** | **Matina 1-6** | 200/200 | 119/119 | 283/283 | 227/227 | 292/292 | 161/161 | 247/247 | 272/272 | 286/286 | 223/223 | 245/245 |
| **Contamana** | **Scavina 12** | 178/194 | 129/139 | 293/293 | 239/241 | 302/322 | 140/146 | 247/249 | 260/266 | 284/284 | 225/225 | 243/257 |
| **Contamana** | **Scavina 6** | 178/194 | 129/139 | 291/291 | 221/221 | 298/306 | 140/149 | 245/249 | 266/272 | 284/290 | 225/225 | 241/243 |
| **Curaray** | **LCTEEN_188** | 178/194 | 139/139 | 287/287 | 215/223 | 300/306 | 132/159 | 247/249 | 260/260 | 284/292 | 225/228 | 250/265 |
| **Curaray** | **LCTEEN_255** | 194/200 | 119/129 | 281/287 | 225/225 | 292/308 | 142/159 | 247/247 | 272/272 | 284/284 | 228/228 | 233/233 |
| **Curaray** | **LCTeen_32** | 194/194 | 139/146 | 287/287 | 215/233 | 300/304 | 146/159 | 249/249 | 260/260 | 292/292 | 225/231 | 235/235 |
| **Curaray** | **LCTEEN_327** | 194/194 | 139/139 | 287/287 | 215/215 | 300/300 | 159/159 | 249/249 | 260/260 | 292/292 | 225/225 | 235/235 |
| **Curaray** | **LCTEEN_36** | 194/197 | 142/142 | 275/275 | 209/209 | 307/320 | 132/142 | 247/249 | 272/272 | 286/290 | 225/225 | 235/235 |
| **Curaray** | **LCTEEN_37** | 194/197 | 119/137 | 283/288 | 211/211 | 294/304 | 140/146 | 245/247 | 260/260 | 284/284 | 225/225 | 235/243 |
| **Curaray** | **LCTEEN_403** | 197/197 | 119/142 | 283/283 | 209/235 | 290/290 | 132/146 | 247/249 | 260/272 | 284/290 | 225/231 | 235/235 |
| **Curaray** | **LCTEEN_189** | 178/194 | 119/139 | 287/287 | 215/223 | 300/306 | 132/159 | 247/249 | 260/272 | 284/292 | 225/228 | 250/265 |
| **Guiana** | **GU114_P** | 197/197 | 119/151 | 283/283 | 231/243 | 304/304 | 145/151 | 245/247 | 272/272 | 284/286 | 225/228 | 245/245 |
| **Guiana** | **GU151_F** | 197/197 | 119/119 | 283/283 | 243/243 | 304/304 | 151/151 | 247/247 | 272/272 | 284/284 | 225/225 | 245/245 |
| **Guiana** | **GU195_P** | 194/197 | 119/119 | 283/283 | 233/233 | 304/304 | 145/151 | 247/249 | 272/272 | 284/292 | 228/228 | 245/245 |
| **Guiana** | **GU219_P** | 197/197 | 119/119 | 283/283 | 243/243 | 304/304 | 151/151 | 247/247 | 272/272 | 284/290 | 228/228 | 245/245 |
| **Guiana** | **GU241_P** | 194/197 | 119/119 | 283/283 | 233/243 | 304/304 | 145/151 | 247/249 | 272/272 | 286/292 | 228/228 | 245/245 |
| **Guiana** | **GU261_P** | 197/197 | 119/119 | 283/283 | 243/243 | 304/304 | 151/151 | 247/247 | 272/272 | 284/284 | 225/228 | 245/245 |
| **Guiana** | **GU277_G** | 197/197 | 119/119 | 283/283 | 243/243 | 304/304 | 151/151 | 247/247 | 272/272 | 284/284 | 225/228 | 245/245 |
| **Guiana** | **GU286** | 197/200 | 119/151 | 283/283 | 231/243 | 304/304 | 151/151 | 245/247 | 272/272 | 286/286 | 225/228 | 245/245 |
| **Guiana** | **GU310_P** | 194/197 | 119/119 | 283/283 | 233/243 | 304/304 | 145/151 | 247/249 | 272/272 | 284/292 | 223/228 | 245/245 |
| **Guiana** | **GU335_P** | 194/197 | 119/119 | 283/283 | 233/243 | 304/304 | 145/151 | 247/249 | 272/272 | 284/292 | 225/228 | 245/245 |
| **Iquitos** | **IMC105** | 194/200 | 119/135 | 283/283 | 225/233 | 302/304 | 133/145 | 245/249 | 260/272 | 286/292 | 228/228 | 235/243 |
| **Iquitos** | **IMC107** | 178/194 | 135/142 | 278/283 | 233/233 | 304/304 | 133/140 | 245/245 | 266/272 | 284/292 | 223/228 | 243/245 |
| **Iquitos** | **IMC2** | 178/178 | 135/142 | 278/278 | 227/227 | 304/306 | 140/151 | 245/249 | 266/272 | 284/290 | 228/228 | 235/243 |
| **Iquitos** | **IMC48** | 194/200 | 119/135 | 283/283 | 225/233 | 302/304 | 130/145 | 245/249 | 260/272 | 286/292 | 223/228 | 243/245 |
| **Iquitos** | **IMC50** | 178/194 | 135/142 | 278/283 | 233/233 | 304/304 | 130/137 | 245/245 | 266/272 | 284/292 | 228/228 | 235/243 |
| **Iquitos** | **IMC55** | 178/194 | 135/142 | 278/283 | 233/233 | 304/304 | 130/137 | 245/245 | 266/272 | 284/292 | 223/228 | 243/245 |
| **Iquitos** | **IMC60** | 194/194 | 135/151 | 283/283 | 233/233 | 304/306 | 133/145 | 247/247 | 272/272 | 284/286 | 223/228 | 233/245 |
| **Iquitos** | **IMC76** | 194/200 | 119/135 | 283/283 | 225/233 | 302/304 | 133/145 | 245/249 | 260/272 | 286/292 | 228/228 | 235/243 |
| **Iquitos** | **IMC98** | 178/178 | 142/151 | 278/278 | 237/237 | 304/306 | 140/145 | 245/247 | 266/272 | 286/286 | 228/228 | 235/243 |
| **Marañón** | **PA141** | 194/200 | 119/135 | 283/283 | 231/231 | 312/312 | 132/146 | 247/247 | 272/272 | 286/286 | 228/228 | 245/245 |
| **Marañón** | **PA151** | 194/200 | 119/135 | 283/283 | 231/231 | 312/312 | 132/146 | 247/247 | 272/272 | 286/286 | 228/228 | 245/245 |
| **Marañón** | **PA16** | 194/194 | 135/135 | 283/283 | 231/231 | 312/312 | 132/146 | 247/247 | 272/272 | 286/286 | 225/228 | 245/250 |
| **Marañón** | **PA30** | 194/194 | 135/135 | 283/283 | 231/231 | 312/312 | 132/146 | 247/247 | 272/272 | 286/286 | 228/228 | 245/245 |
| **Marañón** | **PA32** | 194/194 | 135/135 | 283/283 | 231/231 | 304/304 | 133/139 | 247/247 | 272/272 | 286/286 | 228/228 | 243/243 |
| **Marañón** | **PA39** | 194/200 | 119/135 | 283/283 | 231/231 | 312/312 | 132/145 | 247/249 | 272/272 | 286/286 | 223/228 | 245/245 |
| **Marañón** | **PA7a** | 194/194 | 129/135 | 283/283 | 223/231 | 318/318 | 129/145 | 247/249 | 266/272 | 286/286 | 228/228 | 245/245 |
| **Nacional** | **MO109** | 200/200 | 119/119 | 283/283 | 227/227 | 292/292 | 161/161 | 247/247 | 272/272 | 286/286 | 223/223 | 245/245 |
| **Nacional** | **MO96** | 178/194 | 137/142 | 278/278 | 211/211 | 304/304 | 145/146 | 247/249 | 260/260 | 284/284 | 233/233 | 245/247 |
| **Nanay** | **NA30** | 200/200 | 119/119 | 283/283 | 227/227 | 292/292 | 161/161 | 247/247 | 272/272 | 286/286 | 223/228 | 245/245 |
| **Nanay** | **NA32** | 194/194 | 135/151 | 283/283 | 233/233 | 304/306 | 130/145 | 247/247 | 272/272 | 284/286 | 223/223 | 245/245 |
| **Nanay** | **NA34** | 194/194 | 135/151 | 283/283 | 233/233 | 304/306 | 130/145 | 247/247 | 272/272 | 284/286 | 223/228 | 233/245 |
| **Nanay** | **NA84** | 194/194 | 135/135 | 283/283 | 233/233 | 304/304 | 133/133 | 247/247 | 272/272 | 284/284 | 228/228 | 243/243 |
| **Nanay** | **P10-C** | 194/194 | 119/135 | 283/283 | 233/233 | 304/304 | 133/133 | 247/247 | 272/272 | 286/286 | 228/228 | 243/243 |
| **Nanay** | **P25-A** | 194/194 | 135/135 | 283/283 | 233/233 | 304/304 | 133/133 | 247/247 | 272/272 | 284/284 | 228/228 | 243/243 |
| **Nanay** | **P26** | 194/197 | 119/135 | 283/283 | 233/235 | 304/304 | 133/139 | 247/247 | 272/272 | 292/292 | 228/228 | 243/243 |
| **Nanay** | **P32-A** | 194/197 | 119/135 | 283/283 | 233/235 | 304/304 | 133/139 | 247/247 | 272/272 | 284/292 | 228/228 | 243/243 |
| **Purus** | **LCTEEN_362** | 178/194 | 137/139 | 297/313 | 241/241 | 300/320 | 135/149 | 247/249 | 260/272 | 292/292 | 228/228 | 257/261 |
| **Trinitario** | **GS29** | 194/200 | 119/137 | 280/283 | 227/237 | 292/300 | 134/161 | 247/247 | 260/272 | 286/292 | 223/228 | 239/245 |
| **Trinitario** | **GS77** | 200/200 | 119/119 | 283/283 | 227/227 | 292/292 | 161/161 | 247/247 | 272/272 | 286/286 | 223/228 | 239/245 |
| **Trinitario** | **ICS1** | 200/200 | 119/119 | 283/283 | 227/227 | 292/292 | 161/161 | 247/259 | 272/272 | 286/286 | 223/228 | 239/245 |
| **Trinitario** | **ICS100** | 194/200 | 119/137 | 280/283 | 227/227 | 292/292 | 134/161 | 247/247 | 260/272 | 286/292 | 228/228 | 239/239 |
| **Trinitario** | **ICS15** | 194/200 | 119/137 | 280/283 | 227/239 | 292/300 | 134/161 | 247/247 | 260/272 | 286/292 | 223/228 | 239/245 |
| **Trinitario** | **ICS24** | 200/200 | 119/119 | 283/283 | 227/227 | 292/292 | 161/161 | 247/247 | 272/272 | 286/286 | 223/223 | 245/245 |
| **Trinitario** | **ICS27** | 200/200 | 119/119 | 283/283 | 227/227 | 292/292 | 161/161 | 247/247 | 272/272 | 286/286 | 223/228 | 239/239 |
| **Trinitario** | **ICS40** | 194/194 | 135/151 | 283/283 | 231/237 | 306/306 | 145/145 | 247/247 | 272/272 | 286/286 | 225/228 | 235/243 |
| **Trinitario** | **ICS46** | 194/194 | 135/151 | 283/283 | 231/237 | 306/306 | 145/145 | 247/247 | 272/272 | 286/286 | 225/228 | 235/243 |
| **Trinitario** | **ICS52** | 194/200 | 119/137 | 280/283 | 227/233 | 292/300 | 134/161 | 247/247 | 260/272 | 286/292 | 223/228 | 239/245 |
| **Trinitario** | **ICS53** | 197/197 | 119/151 | 283/283 | 235/239 | 302/306 | 139/145 | 247/247 | 272/272 | 286/292 | 223/228 | 243/245 |
| **Trinitario** | **ICS61** | 178/200 | 119/139 | 283/283 | 227/239 | 292/322 | 140/161 | 247/249 | 266/272 | 284/286 | 225/228 | 241/241 |
| **Trinitario** | **ICS62** | 197/200 | 119/119 | 283/283 | 225/235 | 302/302 | 145/145 | 247/249 | 260/272 | 286/292 | 225/228 | 235/241 |
| **Trinitario** | **ICS67** | 178/197 | 119/139 | 283/283 | 235/239 | 302/302 | 140/140 | 247/249 | 266/272 | 284/292 | 223/225 | 241/245 |
| **Trinitario** | **ICS73** | 178/194 | 135/139 | 283/283 | 233/239 | 304/304 | 140/140 | 245/249 | 266/272 | 284/292 | 223/228 | 245/245 |
| **Trinitario** | **ICS76** | 194/200 | 119/137 | 280/283 | 227/239 | 292/300 | 134/161 | 247/247 | 260/272 | 286/286 | 228/228 | 239/239 |
| **Trinitario** | **ICS77** | 178/200 | 119/139 | 283/283 | 227/239 | 292/322 | 140/161 | 247/249 | 266/272 | 284/286 | 223/225 | 241/245 |
| **Trinitario** | **ICS8** | 200/200 | 119/119 | 283/283 | 227/227 | 292/292 | 161/161 | 247/249 | 272/272 | 286/286 | 223/228 | 239/245 |
| **Trinitario** | **ICS83** | 194/200 | 119/137 | 280/283 | 227/239 | 292/300 | 134/161 | 247/247 | 260/272 | 286/292 | 223/223 | 245/245 |
| **Trinitario** | **UF676** | 194/200 | 119/137 | 283/285 | 217/227 | 292/300 | 134/161 | 247/247 | 260/272 | 286/292 | 223/228 | 239/245 |
| **hybrid** | **EET_103** | 194/200 | 119/139 | 283/287 | 215/227 | 292/300 | 138/161 | 247/249 | 260/272 | 286/292 | 223/233 | 245/245 |
| **hybrid** | **EET_399** | 194/197 | 119/133 | 283/285 | 223/235 | 302/302 | 140/140 | 247/247 | 266/272 | 286/292 | 228/231 | 235/243 |
| **hybrid** | **CCN51** | 200/200 | 119/119 | 283/283 | 225/227 | 292/302 | 145/161 | 247/249 | 260/272 | 286/286 | 228/228 | 235/239 |

Newly references SSR profiles were established for a collection of diverse *T. cacao* clones available from international germplasm collections, and which could be used as standards for prediction of self-compatibility status of cocoa trees.

**Table S4. Relative expression of candidate genes.**

| Gene | SI | | | | | | SC | | | | | |
|---|---|---|---|---|---|---|---|---|---|---|---|---|
| | T2-8h | | T 12-24h | | T 48-72h | | T2-8h | | T 12-24h | | T 48-72h | |
| | RE | SD | RE | SD | RE | SD | RE | SD | RE | SD | RE | SD |
| Tc01_g007220 | 1,79 | 0,6 | 3,11 | 1,57 | 1,57 | 0,36 | 0,85 | 0,65 | 2,46 | 1,34 | 7,75 | 2,69 |
| Tc01_g007270 | 4,60 | 1,340 | 1,87 | 0,685 | 1,38 | 0,651 | 1,57 | 0,757 | 1,62 | 0,685 | 0,71 | 0,651 |
| Tc01_g007290 | 162,75 | 29,458 | 59,68 | 13,878 | 71,50 | 10,080 | 13,89 | 3,219 | 33,79 | 10,080 | 2,87 | 1,340 |
| Tc04_g000160 | 10,44 | 1,450 | 2,67 | 0,380 | 5,64 | 0,780 | 3,70 | 0,520 | 7,66 | 1,070 | 0,88 | 0,120 |
| Tc04_g000170 | 1,13 | 1,008 | 0,88 | 0,311 | 1,20 | 0,757 | 0,50 | 0,486 | 0,16 | 0,311 | 0,16 | 0,311 |
| Tc04_g000190 | 36,39 | 4,967 | 6,40 | 1,070 | 9,23 | 2,382 | 10,87 | 4,967 | 69,01 | 32,424 | 1,95 | 2,382 |
| Tc04_g000230 | 0,37 | 0,559 | 0,03 | 0,023 | 0,67 | 0,681 | 0,17 | 0,113 | 1,37 | 1,070 | 0,18 | 0,023 |
| Tc04_g000240 | 70,43 | 35,604 | 0,99 | 2,578 | 0,70 | 0,509 | 16,54 | 11,450 | 1,50 | 2,578 | 0,29 | 0,509 |
| Tc04_g000260 | 0,02 | 0,037 | 0,01 | 0,006 | 1,58 | 1,000 | 0,03 | 0,041 | 0,13 | 0,252 | 0,05 | 0,088 |
| Tc04_g000300 | 0,16 | 0,205 | 0,38 | 0,224 | 0,08 | 0,077 | 0,16 | 0,205 | 0,39 | 0,409 | 0,23 | 0,224 |
| Tc04_g000320 | 2,06 | 1,263 | 2,89 | 1,993 | 2,25 | 1,263 | 0,71 | 0,739 | 14,59 | 7,789 | 1,97 | 1,993 |
| Tc04_g000330 | 1,19 | 1,924 | 2,30 | 2,497 | 1,25 | 0,739 | 1,90 | 1,823 | 7,39 | 2,952 | 1,34 | 0,739 |

The relative expression (RE) and standard deviation (SD) of candidate genes identified in the CH1 and CH4 chromosome regions was calculated at several times (2-8h, 12-24h and 48-72h) after self incompatible (SI) and self-compatible (SC) pollinations made on the SI clone Scavina 6, and with 3 repetitions for each condition. Candidate gene expression was first normalized with two housekeeping genes and their relative expression calculated in relation to un-pollinated ovules.

**Table S5. Allelic frequencies of SSR markers located in CH1 and CH4 regions in the several *T. cacao* genetic groups and populations studied.**

| | *T. cacao* genetic groups | | | | | | | | | | Populations | | | | |
|---|---|---|---|---|---|---|---|---|---|---|---|---|---|---|---|
| LOCUS /alleles | Amelonado | Contamana | Criollo | Curaray | Guiana | Iquitos | Maranon | Nacional | Nanay | Purus | BR | CAM-cult | CAM-Brd | ECU | TR |
| **mSi7/ 7 all.** | | | | | | | | | | | | | | | |
| *(N)* | *14* | *6* | *5* | *10* | *12* | *13* | *13* | *11* | *16* | *8* | *225* | *139* | *125* | *94* | *58* |
| **156** | 0,00 | 0,00 | 0,00 | 0,00 | 0,00 | 0,00 | 0,00 | 0,00 | 0,00 | 0,06 | 0,02 | 0,01 | 0,00 | 0,00 | 0,00 |
| **175** | 0,00 | 0,00 | 0,00 | 0,00 | 0,00 | 0,00 | 0,00 | 0,00 | 0,00 | 0,13 | 0,00 | 0,00 | 0,00 | 0,00 | 0,00 |
| **177** | 0,00 | 0,00 | 0,00 | 0,00 | 0,00 | 0,00 | 0,04 | 0,00 | 0,00 | 0,00 | 0,00 | 0,01 | 0,00 | 0,02 | 0,00 |
| **178** | 0,00 | 0,33 | 0,00 | 0,25 | 0,00 | 0,46 | 0,00 | 0,14 | 0,06 | 0,19 | 0,08 | 0,00 | 0,02 | 0,06 | 0,08 |
| **194** | 0,00 | 0,50 | 1,00 | 0,50 | 0,17 | 0,35 | 0,69 | 0,73 | 0,66 | 0,44 | 0,38 | 0,53 | 0,54 | 0,48 | 0,31 |
| **197** | 0,00 | 0,00 | 0,00 | 0,20 | 0,79 | 0,00 | 0,00 | 0,00 | 0,22 | 0,13 | 0,10 | 0,01 | 0,00 | 0,01 | 0,06 |
| **200** | 1,00 | 0,17 | 0,00 | 0,05 | 0,04 | 0,19 | 0,27 | 0,14 | 0,06 | 0,06 | 0,42 | 0,45 | 0,44 | 0,43 | 0,55 |
| **mSI_35/17 all.** | | | | | | | | | | | | | | | |
| *(N)* | *13* | *6* | *5* | *9* | *12* | *13* | *13* | *10* | *18* | *7* | *214* | *124* | *125* | *92* | *58* |
| **119** | 1,00 | 0,08 | 0,00 | 0,22 | 0,88 | 0,15 | 0,31 | 0,15 | 0,39 | 0,14 | 0,54 | 0,44 | 0,37 | 0,43 | 0,60 |
| **125** | 0,00 | 0,00 | 0,00 | 0,00 | 0,00 | 0,00 | 0,00 | 0,00 | 0,06 | 0,00 | 0,04 | 0,00 | 0,00 | 0,00 | 0,00 |
| **127** | 0,00 | 0,00 | 0,00 | 0,00 | 0,00 | 0,00 | 0,00 | 0,05 | 0,00 | 0,07 | 0,00 | 0,00 | 0,00 | 0,00 | 0,00 |
| **129** | 0,00 | 0,33 | 0,00 | 0,11 | 0,00 | 0,00 | 0,04 | 0,10 | 0,03 | 0,07 | 0,11 | 0,16 | 0,05 | 0,00 | 0,03 |
| **131** | 0,00 | 0,00 | 0,00 | 0,00 | 0,00 | 0,00 | 0,00 | 0,00 | 0,00 | 0,07 | 0,00 | 0,00 | 0,00 | 0,00 | 0,00 |
| **133** | 0,00 | 0,00 | 0,00 | 0,00 | 0,00 | 0,00 | 0,00 | 0,05 | 0,00 | 0,00 | 0,03 | 0,00 | 0,00 | 0,01 | 0,00 |
| **135** | 0,00 | 0,08 | 0,00 | 0,00 | 0,00 | 0,35 | 0,62 | 0,00 | 0,42 | 0,07 | 0,04 | 0,04 | 0,34 | 0,00 | 0,04 |
| **137** | 0,00 | 0,00 | 1,00 | 0,06 | 0,00 | 0,00 | 0,00 | 0,05 | 0,06 | 0,29 | 0,10 | 0,22 | 0,08 | 0,02 | 0,15 |
| **139** | 0,00 | 0,33 | 0,00 | 0,33 | 0,00 | 0,00 | 0,00 | 0,50 | 0,00 | 0,07 | 0,07 | 0,00 | 0,00 | 0,48 | 0,09 |
| **140** | 0,00 | 0,00 | 0,00 | 0,06 | 0,00 | 0,00 | 0,00 | 0,05 | 0,00 | 0,00 | 0,04 | 0,00 | 0,00 | 0,07 | 0,00 |
| **142** | 0,00 | 0,00 | 0,00 | 0,17 | 0,00 | 0,31 | 0,00 | 0,05 | 0,00 | 0,00 | 0,00 | 0,00 | 0,00 | 0,00 | 0,00 |
| **144** | 0,00 | 0,00 | 0,00 | 0,00 | 0,00 | 0,00 | 0,00 | 0,00 | 0,00 | 0,00 | 0,00 | 0,00 | 0,00 | 0,00 | 0,01 |
| **146** | 0,00 | 0,00 | 0,00 | 0,06 | 0,00 | 0,00 | 0,00 | 0,00 | 0,00 | 0,07 | 0,00 | 0,00 | 0,02 | 0,00 | 0,00 |
| **148** | 0,00 | 0,00 | 0,00 | 0,00 | 0,00 | 0,00 | 0,00 | 0,00 | 0,00 | 0,07 | 0,00 | 0,00 | 0,00 | 0,00 | 0,00 |
| **151** | 0,00 | 0,17 | 0,00 | 0,00 | 0,13 | 0,19 | 0,00 | 0,00 | 0,06 | 0,00 | 0,01 | 0,13 | 0,14 | 0,00 | 0,08 |
| **156** | 0,00 | 0,00 | 0,00 | 0,00 | 0,00 | 0,00 | 0,00 | 0,00 | 0,00 | 0,07 | 0,00 | 0,00 | 0,00 | 0,00 | 0,00 |
| **158** | 0,00 | 0,00 | 0,00 | 0,00 | 0,00 | 0,00 | 0,04 | 0,00 | 0,00 | 0,00 | 0,01 | 0,00 | 0,00 | 0,00 | 0,00 |
| **mSI_303/19 all.** | | | | | | | | | | | | | | | |
| *(N)* | *11* | *5* | *5* | *10* | *12* | *12* | *11* | *12* | *13* | *8* | *251* | *145* | *124* | *94* | *56* |
| **207** | 0,00 | 0,00 | 0,00 | 0,00 | 0,00 | 0,00 | 0,00 | 0,00 | 0,00 | 0,00 | 0,00 | 0,00 | 0,00 | 0,00 | 0,00 |
| **209** | 0,00 | 0,00 | 0,00 | 0,30 | 0,00 | 0,00 | 0,00 | 0,00 | 0,00 | 0,00 | 0,00 | 0,00 | 0,00 | 0,00 | 0,00 |
| **211** | 0,00 | 0,00 | 0,00 | 0,10 | 0,00 | 0,00 | 0,00 | 0,21 | 0,00 | 0,00 | 0,10 | 0,04 | 0,01 | 0,00 | 0,00 |
| **215** | 0,00 | 0,00 | 0,00 | 0,25 | 0,00 | 0,00 | 0,00 | 0,29 | 0,00 | 0,06 | 0,03 | 0,00 | 0,00 | 0,39 | 0,02 |
| **217** | 0,00 | 0,00 | 1,00 | 0,00 | 0,00 | 0,00 | 0,00 | 0,00 | 0,00 | 0,00 | 0,01 | 0,10 | 0,00 | 0,00 | 0,04 |
| **221** | 0,00 | 0,20 | 0,00 | 0,00 | 0,00 | 0,00 | 0,00 | 0,00 | 0,00 | 0,00 | 0,08 | 0,02 | 0,03 | 0,01 | 0,01 |
| **223** | 0,00 | 0,00 | 0,00 | 0,15 | 0,00 | 0,00 | 0,05 | 0,25 | 0,00 | 0,19 | 0,09 | 0,13 | 0,00 | 0,08 | 0,00 |
| **225** | 0,00 | 0,00 | 0,00 | 0,10 | 0,00 | 0,17 | 0,05 | 0,04 | 0,00 | 0,06 | 0,07 | 0,04 | 0,03 | 0,01 | 0,03 |
| **227** | 0,91 | 0,00 | 0,00 | 0,00 | 0,00 | 0,08 | 0,00 | 0,08 | 0,08 | 0,13 | 0,35 | 0,31 | 0,27 | 0,40 | 0,47 |
| **229** | 0,09 | 0,00 | 0,00 | 0,00 | 0,00 | 0,00 | 0,00 | 0,00 | 0,00 | 0,00 | 0,00 | 0,00 | 0,02 | 0,00 | 0,03 |
| **231** | 0,00 | 0,00 | 0,00 | 0,00 | 0,13 | 0,00 | 0,91 | 0,00 | 0,00 | 0,13 | 0,00 | 0,05 | 0,39 | 0,01 | 0,04 |
| **233** | 0,00 | 0,20 | 0,00 | 0,05 | 0,21 | 0,46 | 0,00 | 0,04 | 0,81 | 0,13 | 0,18 | 0,21 | 0,25 | 0,00 | 0,04 |
| **235** | 0,00 | 0,00 | 0,00 | 0,05 | 0,00 | 0,00 | 0,00 | 0,00 | 0,12 | 0,00 | 0,02 | 0,00 | 0,00 | 0,01 | 0,04 |
| **237** | 0,00 | 0,10 | 0,00 | 0,00 | 0,00 | 0,29 | 0,00 | 0,04 | 0,00 | 0,13 | 0,03 | 0,04 | 0,00 | 0,01 | 0,12 |
| **239** | 0,00 | 0,30 | 0,00 | 0,00 | 0,00 | 0,00 | 0,00 | 0,00 | 0,00 | 0,00 | 0,03 | 0,06 | 0,00 | 0,02 | 0,14 |
| **241** | 0,00 | 0,20 | 0,00 | 0,00 | 0,00 | 0,00 | 0,00 | 0,00 | 0,00 | 0,19 | 0,00 | 0,00 | 0,00 | 0,00 | 0,03 |
| **243** | 0,00 | 0,00 | 0,00 | 0,00 | 0,67 | 0,00 | 0,00 | 0,04 | 0,00 | 0,00 | 0,00 | 0,00 | 0,00 | 0,07 | 0,00 |
| **245** | 0,00 | 0,00 | 0,00 | 0,00 | 0,00 | 0,00 | 0,00 | 0,00 | 0,00 | 0,00 | 0,00 | 0,00 | 0,00 | 0,00 | 0,00 |
| **247** | 0,00 | 0,00 | 0,00 | 0,00 | 0,00 | 0,00 | 0,00 | 0,00 | 0,00 | 0,00 | 0,00 | 0,00 | 0,00 | 0,00 | 0,00 |
| **mSI_458/24 all.** | | | | | | | | | | | | | | | |
| *(N)* | *11* | *5* | *5* | *10* | *11* | *12* | *11* | *11* | *13* | *8* | *252* | *139* | *120* | *94* | *55* |
| **286** | 0,00 | 0,00 | 0,00 | 0,00 | 0,00 | 0,00 | 0,00 | 0,00 | 0,00 | 0,13 | 0,00 | 0,00 | 0,00 | 0,00 | 0,00 |
| **290** | 0,00 | 0,00 | 0,00 | 0,10 | 0,00 | 0,00 | 0,00 | 0,00 | 0,00 | 0,06 | 0,02 | 0,00 | 0,00 | 0,00 | 0,00 |
| **292** | 1,00 | 0,00 | 0,00 | 0,05 | 0,00 | 0,00 | 0,00 | 0,09 | 0,08 | 0,31 | 0,36 | 0,35 | 0,39 | 0,45 | 0,51 |
| **294** | 0,00 | 0,00 | 0,00 | 0,05 | 0,00 | 0,00 | 0,00 | 0,00 | 0,00 | 0,00 | 0,01 | 0,00 | 0,00 | 0,00 | 0,00 |
| **296** | 0,00 | 0,00 | 0,00 | 0,00 | 0,00 | 0,00 | 0,00 | 0,00 | 0,15 | 0,06 | 0,08 | 0,00 | 0,00 | 0,00 | 0,00 |
| **298** | 0,00 | 0,10 | 0,00 | 0,00 | 0,00 | 0,00 | 0,00 | 0,00 | 0,00 | 0,00 | 0,00 | 0,00 | 0,00 | 0,00 | 0,00 |
| **300** | 0,00 | 0,00 | 1,00 | 0,25 | 0,00 | 0,00 | 0,00 | 0,36 | 0,00 | 0,06 | 0,07 | 0,23 | 0,07 | 0,41 | 0,16 |
| **302** | 0,00 | 0,20 | 0,00 | 0,00 | 0,00 | 0,17 | 0,00 | 0,00 | 0,00 | 0,00 | 0,14 | 0,05 | 0,06 | 0,03 | 0,11 |
| **304** | 0,00 | 0,10 | 0,00 | 0,10 | 1,00 | 0,58 | 0,18 | 0,27 | 0,69 | 0,19 | 0,18 | 0,04 | 0,12 | 0,00 | 0,04 |
| **306** | 0,00 | 0,20 | 0,00 | 0,15 | 0,00 | 0,25 | 0,00 | 0,09 | 0,08 | 0,00 | 0,12 | 0,19 | 0,16 | 0,08 | 0,12 |
| **307** | 0,00 | 0,00 | 0,00 | 0,05 | 0,00 | 0,00 | 0,00 | 0,00 | 0,00 | 0,00 | 0,00 | 0,00 | 0,00 | 0,00 | 0,00 |
| **308** | 0,00 | 0,00 | 0,00 | 0,05 | 0,00 | 0,00 | 0,00 | 0,00 | 0,00 | 0,13 | 0,00 | 0,00 | 0,00 | 0,00 | 0,01 |
| **310** | 0,00 | 0,00 | 0,00 | 0,00 | 0,00 | 0,00 | 0,00 | 0,00 | 0,00 | 0,00 | 0,01 | 0,00 | 0,00 | 0,01 | 0,00 |
| **312** | 0,00 | 0,00 | 0,00 | 0,00 | 0,00 | 0,00 | 0,73 | 0,09 | 0,00 | 0,00 | 0,00 | 0,00 | 0,18 | 0,01 | 0,02 |
| **314** | 0,00 | 0,00 | 0,00 | 0,00 | 0,00 | 0,00 | 0,00 | 0,00 | 0,00 | 0,00 | 0,01 | 0,00 | 0,00 | 0,00 | 0,00 |
| **316** | 0,00 | 0,00 | 0,00 | 0,00 | 0,00 | 0,00 | 0,00 | 0,00 | 0,00 | 0,00 | 0,00 | 0,00 | 0,00 | 0,00 | 0,00 |
| **318** | 0,00 | 0,00 | 0,00 | 0,00 | 0,00 | 0,00 | 0,09 | 0,09 | 0,00 | 0,00 | 0,00 | 0,09 | 0,00 | 0,00 | 0,00 |
| **320** | 0,00 | 0,00 | 0,00 | 0,20 | 0,00 | 0,00 | 0,00 | 0,00 | 0,00 | 0,06 | 0,00 | 0,00 | 0,00 | 0,00 | 0,00 |
| **321** | 0,00 | 0,00 | 0,00 | 0,00 | 0,00 | 0,00 | 0,00 | 0,00 | 0,00 | 0,00 | 0,00 | 0,00 | 0,00 | 0,00 | 0,00 |
| **322** | 0,00 | 0,30 | 0,00 | 0,00 | 0,00 | 0,00 | 0,00 | 0,00 | 0,00 | 0,00 | 0,00 | 0,00 | 0,00 | 0,01 | 0,04 |
| **323** | 0,00 | 0,00 | 0,00 | 0,00 | 0,00 | 0,00 | 0,00 | 0,00 | 0,00 | 0,00 | 0,00 | 0,00 | 0,00 | 0,00 | 0,00 |
| **324** | 0,00 | 0,10 | 0,00 | 0,00 | 0,00 | 0,00 | 0,00 | 0,00 | 0,00 | 0,00 | 0,00 | 0,00 | 0,00 | 0,00 | 0,00 |
| **325** | 0,00 | 0,00 | 0,00 | 0,00 | 0,00 | 0,00 | 0,00 | 0,00 | 0,00 | 0,00 | 0,00 | 0,00 | 0,00 | 0,00 | 0,00 |
| **330** | 0,00 | 0,00 | 0,00 | 0,00 | 0,00 | 0,00 | 0,00 | 0,00 | 0,00 | 0,00 | 0,00 | 0,05 | 0,02 | 0,00 | 0,00 |

**Table S5 (continued)**

| | *T. cacao* genetic groups | | | | | | | | | | Populations | | | | |
|---|---|---|---|---|---|---|---|---|---|---|---|---|---|---|---|
| **LOCUS /alleles** | **Amelonado** | **Contamana** | **Criollo** | **Curaray** | **Guiana** | **Iquitos** | **Maranon** | **Nacional** | **Nanay** | **Purus** | **BR** | **CAM-cult** | **CAM-Brd** | **ECU** | **TR** |
| **mSI_460/ 22 all.** | | | | | | | | | | | | | | | |
| ***(N)*** | *11* | *5* | *5* | *10* | *11* | *12* | *11* | *12* | *13* | *8* | *262* | *147* | *123* | *95* | *52* |
| **129** | 0,00 | 0,00 | 0,00 | 0,00 | 0,00 | 0,00 | 0,05 | 0,00 | 0,00 | 0,06 | 0,00 | 0,00 | 0,13 | 0,00 | 0,00 |
| **130** | 0,00 | 0,00 | 0,00 | 0,00 | 0,00 | 0,17 | 0,00 | 0,00 | 0,08 | 0,00 | 0,00 | 0,00 | 0,06 | 0,00 | 0,00 |
| **131** | 0,00 | 0,00 | 0,40 | 0,00 | 0,00 | 0,00 | 0,00 | 0,00 | 0,00 | 0,06 | 0,00 | 0,00 | 0,08 | 0,00 | 0,00 |
| **132** | 0,00 | 0,00 | 0,00 | 0,40 | 0,00 | 0,00 | 0,45 | 0,17 | 0,08 | 0,06 | 0,03 | 0,10 | 0,00 | 0,08 | 0,02 |
| **133** | 0,00 | 0,10 | 0,00 | 0,00 | 0,00 | 0,13 | 0,05 | 0,00 | 0,42 | 0,00 | 0,05 | 0,00 | 0,00 | 0,00 | 0,00 |
| **134** | 0,00 | 0,00 | 0,60 | 0,00 | 0,00 | 0,00 | 0,00 | 0,00 | 0,00 | 0,06 | 0,08 | 0,26 | 0,00 | 0,01 | 0,15 |
| **135** | 0,00 | 0,00 | 0,00 | 0,00 | 0,00 | 0,00 | 0,00 | 0,00 | 0,00 | 0,06 | 0,00 | 0,00 | 0,02 | 0,00 | 0,00 |
| **136** | 0,00 | 0,00 | 0,00 | 0,00 | 0,00 | 0,00 | 0,00 | 0,08 | 0,00 | 0,00 | 0,04 | 0,00 | 0,00 | 0,08 | 0,00 |
| **137** | 0,00 | 0,00 | 0,00 | 0,00 | 0,00 | 0,08 | 0,00 | 0,00 | 0,00 | 0,00 | 0,00 | 0,00 | 0,00 | 0,00 | 0,00 |
| **138** | 0,00 | 0,00 | 0,00 | 0,00 | 0,00 | 0,00 | 0,00 | 0,29 | 0,00 | 0,00 | 0,03 | 0,00 | 0,00 | 0,40 | 0,02 |
| **139** | 0,00 | 0,00 | 0,00 | 0,00 | 0,00 | 0,00 | 0,05 | 0,00 | 0,12 | 0,00 | 0,02 | 0,00 | 0,00 | 0,00 | 0,01 |
| **140** | 0,00 | 0,40 | 0,00 | 0,05 | 0,00 | 0,21 | 0,00 | 0,17 | 0,00 | 0,00 | 0,11 | 0,01 | 0,00 | 0,02 | 0,12 |
| **142** | 0,00 | 0,00 | 0,00 | 0,10 | 0,00 | 0,00 | 0,00 | 0,00 | 0,00 | 0,06 | 0,00 | 0,00 | 0,00 | 0,00 | 0,00 |
| **145** | 0,00 | 0,10 | 0,00 | 0,00 | 0,27 | 0,38 | 0,18 | 0,08 | 0,08 | 0,19 | 0,06 | 0,27 | 0,32 | 0,02 | 0,10 |
| **146** | 0,00 | 0,20 | 0,00 | 0,15 | 0,00 | 0,00 | 0,18 | 0,08 | 0,00 | 0,00 | 0,07 | 0,03 | 0,09 | 0,00 | 0,05 |
| **149** | 0,09 | 0,10 | 0,00 | 0,00 | 0,00 | 0,00 | 0,00 | 0,04 | 0,00 | 0,06 | 0,06 | 0,25 | 0,16 | 0,00 | 0,02 |
| **151** | 0,00 | 0,00 | 0,00 | 0,00 | 0,73 | 0,04 | 0,00 | 0,00 | 0,00 | 0,13 | 0,01 | 0,00 | 0,00 | 0,00 | 0,00 |
| **152** | 0,00 | 0,10 | 0,00 | 0,00 | 0,00 | 0,00 | 0,05 | 0,00 | 0,00 | 0,00 | 0,00 | 0,00 | 0,00 | 0,00 | 0,00 |
| **155** | 0,00 | 0,00 | 0,00 | 0,00 | 0,00 | 0,00 | 0,00 | 0,00 | 0,15 | 0,00 | 0,11 | 0,00 | 0,00 | 0,00 | 0,00 |
| **157** | 0,00 | 0,00 | 0,00 | 0,00 | 0,00 | 0,00 | 0,00 | 0,00 | 0,00 | 0,06 | 0,00 | 0,00 | 0,00 | 0,00 | 0,00 |
| **159** | 0,27 | 0,00 | 0,00 | 0,30 | 0,00 | 0,00 | 0,00 | 0,00 | 0,00 | 0,19 | 0,00 | 0,00 | 0,02 | 0,00 | 0,08 |
| **161** | 0,64 | 0,00 | 0,00 | 0,00 | 0,00 | 0,00 | 0,00 | 0,08 | 0,08 | 0,00 | 0,32 | 0,09 | 0,11 | 0,40 | 0,44 |
| **mSI_103/ 6 all.** | | | | | | | | | | | | | | | |
| ***(N)*** | *11* | *5* | *5* | *10* | *11* | *12* | *11* | *10* | *13* | *8* | *245* | *116* | *124* | *91* | *53* |
| **223** | 0,73 | 0,20 | 0,00 | 0,00 | 0,05 | 0,21 | 0,05 | 0,25 | 0,15 | 0,00 | 0,26 | 0,53 | 0,67 | 0,38 | 0,41 |
| **225** | 0,00 | 0,80 | 0,00 | 0,70 | 0,41 | 0,00 | 0,05 | 0,00 | 0,00 | 0,13 | 0,18 | 0,05 | 0,07 | 0,00 | 0,12 |
| **228** | 0,27 | 0,00 | 1,00 | 0,20 | 0,55 | 0,79 | 0,91 | 0,05 | 0,85 | 0,81 | 0,47 | 0,41 | 0,25 | 0,10 | 0,45 |
| **229** | 0,00 | 0,00 | 0,00 | 0,00 | 0,00 | 0,00 | 0,00 | 0,00 | 0,00 | 0,06 | 0,00 | 0,00 | 0,00 | 0,00 | 0,00 |
| **231** | 0,00 | 0,00 | 0,00 | 0,10 | 0,00 | 0,00 | 0,00 | 0,05 | 0,00 | 0,00 | 0,03 | 0,01 | 0,01 | 0,01 | 0,00 |
| **233** | 0,00 | 0,00 | 0,00 | 0,00 | 0,00 | 0,00 | 0,00 | 0,65 | 0,00 | 0,00 | 0,06 | 0,00 | 0,00 | 0,51 | 0,02 |
| **mSI_107/ 16 all.** | | | | | | | | | | | | | | | |
| ***(N)*** | *11* | *5* | *5* | *10* | *11* | *12* | *11* | *12* | *13* | *7* | *246* | *141* | *125* | *93* | *51* |
| **233** | 0,00 | 0,00 | 0,00 | 0,10 | 0,00 | 0,04 | 0,00 | 0,00 | 0,04 | 0,00 | 0,00 | 0,00 | 0,00 | 0,00 | 0,00 |
| **235** | 0,00 | 0,00 | 0,00 | 0,65 | 0,00 | 0,29 | 0,00 | 0,00 | 0,00 | 0,07 | 0,22 | 0,02 | 0,00 | 0,03 | 0,07 |
| **237** | 0,00 | 0,00 | 0,00 | 0,00 | 0,00 | 0,00 | 0,00 | 0,00 | 0,00 | 0,57 | 0,02 | 0,00 | 0,00 | 0,00 | 0,00 |
| **239** | 0,14 | 0,00 | 1,00 | 0,00 | 0,00 | 0,00 | 0,00 | 0,04 | 0,00 | 0,00 | 0,13 | 0,26 | 0,00 | 0,09 | 0,25 |
| **241** | 0,00 | 0,10 | 0,00 | 0,00 | 0,00 | 0,00 | 0,00 | 0,00 | 0,00 | 0,00 | 0,06 | 0,00 | 0,00 | 0,00 | 0,08 |
| **243** | 0,09 | 0,30 | 0,00 | 0,05 | 0,00 | 0,46 | 0,09 | 0,00 | 0,54 | 0,14 | 0,20 | 0,07 | 0,12 | 0,01 | 0,13 |
| **245** | 0,50 | 0,20 | 0,00 | 0,00 | 1,00 | 0,21 | 0,86 | 0,50 | 0,19 | 0,07 | 0,30 | 0,66 | 0,88 | 0,66 | 0,43 |
| **247** | 0,14 | 0,00 | 0,00 | 0,00 | 0,00 | 0,00 | 0,00 | 0,29 | 0,08 | 0,00 | 0,06 | 0,00 | 0,01 | 0,18 | 0,04 |
| **249** | 0,14 | 0,00 | 0,00 | 0,00 | 0,00 | 0,00 | 0,00 | 0,00 | 0,00 | 0,00 | 0,00 | 0,00 | 0,00 | 0,03 | 0,00 |
| **250** | 0,00 | 0,00 | 0,00 | 0,10 | 0,00 | 0,00 | 0,05 | 0,00 | 0,00 | 0,00 | 0,00 | 0,00 | 0,00 | 0,00 | 0,00 |
| **255** | 0,00 | 0,00 | 0,00 | 0,00 | 0,00 | 0,00 | 0,00 | 0,04 | 0,00 | 0,00 | 0,00 | 0,00 | 0,00 | 0,00 | 0,00 |
| **256** | 0,00 | 0,00 | 0,00 | 0,00 | 0,00 | 0,00 | 0,00 | 0,08 | 0,00 | 0,00 | 0,00 | 0,00 | 0,00 | 0,00 | 0,00 |
| **257** | 0,00 | 0,40 | 0,00 | 0,00 | 0,00 | 0,00 | 0,00 | 0,04 | 0,00 | 0,07 | 0,00 | 0,00 | 0,00 | 0,01 | 0,00 |
| **259** | 0,00 | 0,00 | 0,00 | 0,00 | 0,00 | 0,00 | 0,00 | 0,00 | 0,00 | 0,00 | 0,00 | 0,00 | 0,00 | 0,00 | 0,00 |
| **261** | 0,00 | 0,00 | 0,00 | 0,00 | 0,00 | 0,00 | 0,00 | 0,00 | 0,15 | 0,07 | 0,00 | 0,00 | 0,00 | 0,00 | 0,00 |
| **265** | 0,00 | 0,00 | 0,00 | 0,10 | 0,00 | 0,00 | 0,00 | 0,00 | 0,00 | 0,00 | 0,00 | 0,00 | 0,00 | 0,00 | 0,00 |

Number of alleles and allelic frequencies, in the several *T. cacao* genetic groups and populations studied, for the most polymorphic markers of CH1 and CH4 regions used to make predictions for self-compatibility trait. BR: Brazilian population; CAM-cult: cultivated population from Cameroun; CAM-brd: breeding population from Cameroun; ECU: Ecuadorian population; TR: Trinitario population (CRC).

**Table S6. Significant predictions of self-compatibility/incompatibility based on genotype presence.**

| marker1 | marker2 | genotype | S_0 | S_1 | N_0 | N_1 | N | XP2_FISH | value(Fishe | ProbSIYs | ProbSCYs |
|---|---|---|---|---|---|---|---|---|---|---|---|
| **mSI_107** | | 239/245 | 32 | 67 | 368 | 195 | 662 | 0,00000 | 0,00000 | 0,32324 | 0,67676 |
| **mSI_303** | | 227/227 | 17 | 57 | 389 | 213 | 676 | 0,00000 | 0,00000 | 0,22973 | 0,77027 |
| **mSI_303** | | 215/227 | 18 | 42 | 388 | 228 | 676 | 0,00000 | 0,00023 | 0,30000 | 0,70000 |
| **mSI_303** | | 223/227 | 12 | 25 | 394 | 245 | 676 | 0,00079 | 0,04032 | 0,32433 | 0,67567 |
| **mSI_303** | | 225/227 | 1 | 14 | 405 | 256 | 676 | 0,00002 | 0,00252 | 0,06668 | **0,93332** |
| **mSI_35** | | 119/119 | 28 | 86 | 338 | 168 | 620 | 0,00000 | 0,00000 | 0,24561 | 0,75439 |
| **mSI_35** | | 119/139 | 36 | 51 | 330 | 203 | 620 | 0,00039 | 0,02514 | 0,41380 | 0,58620 |
| **mSI_458** | | 292/292 | 37 | 81 | 361 | 187 | 666 | 0,00000 | 0,00000 | 0,31356 | 0,68644 |
| **mSI_458** | | 292/300 | 38 | 54 | 360 | 214 | 666 | 0,00014 | 0,01138 | 0,41305 | 0,58695 |
| **mSI_458** | | 292/318 | 0 | 9 | 398 | 259 | 666 | 0,00026 | 0,01771 | 0,00000 | **1,00000** |
| **mSI_460** | | 161/161 | 0 | 44 | 412 | 229 | 685 | 0,00000 | 0,00000 | 0,00000 | **1,00000** |
| **mSI_460** | | 138/161 | 18 | 46 | 394 | 227 | 685 | 0,00000 | 0,00002 | 0,28126 | 0,71874 |
| **mSI_460** | | 132/149 | 1 | 11 | 411 | 262 | 685 | 0,00028 | 0,01899 | 0,08334 | **0,91667** |
| **mSI_7** | | 200/200 | 49 | 88 | 348 | 163 | 648 | 0,00000 | 0,00000 | 0,35770 | **0,64230** |
| **mSI_2** | | 280/283 | 35 | 6 | 253 | 193 | 487 | 0,00022 | 0,01678 | **0,85362** | 0,14638 |
| **mSI_303** | | 231/233 | 23 | 0 | 383 | 270 | 676 | 0,00001 | 0,00127 | **1,00000** | 0,00000 |
| **mSI_303** | | 231/231 | 24 | 0 | 382 | 270 | 676 | 0,00001 | 0,00078 | **1,00000** | 0,00000 |
| **mSI_35** | | 135/151 | 18 | 1 | 348 | 253 | 620 | 0,00066 | 0,03588 | **0,94732** | 0,05268 |
| **mSI_35** | | 119/137 | 55 | 16 | 311 | 238 | 620 | 0,00077 | 0,03938 | 0,77465 | 0,22535 |
| **mSI_458** | | 306/306 | 26 | 2 | 372 | 266 | 666 | 0,00011 | 0,00923 | **0,92856** | 0,07144 |
| **mSI_460** | | 155/161 | 22 | 1 | 390 | 272 | 685 | 0,00014 | 0,01110 | **0,95652** | 0,04348 |
| **mSI_460** | | 145/146 | 24 | 2 | 388 | 271 | 685 | 0,00034 | 0,02264 | **0,92307** | 0,07693 |
| **mSI_7** | | 194/197 | 22 | 1 | 375 | 250 | 648 | 0,00026 | 0,01828 | **0,95652** | 0,04348 |
| **mSI_7** | | 194/194 | 107 | 36 | 290 | 215 | 648 | 0,00014 | 0,01154 | 0,74825 | 0,25175 |

**Table S6 (continued)**

| marker1 | marker2 | genotype | S_0 | S_1 | N_0 | N_1 | N | XP2_FISH | q-value(Fisher) | ProbSIYs | ProbSCYs |
|---|---|---|---|---|---|---|---|---|---|---|---|
| **mSI_103** | **mSI_35** | 223/223 119/119 | 1 | 13 | 387 | 234 | 635 | 0,00003 | 0,00393 | 0,07143 | **0,92857** |
| **mSI_107** | **mSI_2** | 239/245 283/283 | 5 | 31 | 395 | 231 | 662 | 0,00000 | 0,00000 | 0,13892 | **0,86108** |
| **mSI_107** | **mSI_303** | 239/245 227/227 | 1 | 22 | 399 | 240 | 662 | 0,00000 | 0,00000 | 0,04356 | **0,95644** |
| **mSI_107** | **mSI_315** | 239/245 247/249 | 3 | 14 | 397 | 248 | 662 | 0,00050 | 0,03081 | 0,17647 | **0,82353** |
| **mSI_107** | **mSI_35** | 239/245 119/119 | 0 | 27 | 400 | 235 | 662 | 0,00000 | 0,00000 | 0,00000 | **1,00000** |
| **mSI_107** | **mSI_411** | 239/239 286/286 | 0 | 8 | 400 | 254 | 662 | 0,00056 | 0,03370 | 0,00000 | **1,00000** |
| **mSI_107** | **mSI_411** | 239/245 286/286 | 4 | 30 | 396 | 232 | 662 | 0,00000 | 0,00000 | 0,11775 | **0,88225** |
| **mSI_107** | **mSI_458** | 235/245 292/292 | 0 | 9 | 400 | 253 | 662 | 0,00022 | 0,01678 | 0,00000 | **1,00000** |
| **mSI_107** | **mSI_458** | 239/245 292/292 | 1 | 26 | 399 | 236 | 662 | 0,00000 | 0,00000 | 0,03704 | **0,96296** |
| **mSI_107** | **mSI_460** | 239/245 149/161 | 1 | 11 | 399 | 251 | 662 | 0,00025 | 0,01771 | 0,08333 | **0,91667** |
| **mSI_107** | **mSI_7** | 239/245 200/200 | 5 | 30 | 395 | 232 | 662 | 0,00000 | 0,00000 | 0,14289 | **0,85711** |
| **mSI_303** | **mSI_2** | 227/227 283/283 | 6 | 44 | 400 | 226 | 676 | 0,00000 | 0,00000 | 0,12000 | **0,88000** |
| **mSI_303** | **mSI_35** | 227/227 119/119 | 1 | 53 | 405 | 217 | 676 | 0,00000 | 0,00000 | 0,01854 | **0,98146** |
| **mSI_303** | **mSI_35** | 223/227 119/129 | 1 | 11 | 405 | 259 | 676 | 0,00028 | 0,01912 | 0,08334 | **0,91667** |
| **mSI_303** | **mSI_408** | 223/227 266/272 | 4 | 20 | 402 | 250 | 676 | 0,00002 | 0,00199 | 0,16667 | **0,83333** |
| **mSI_303** | **mSI_411** | 227/227 286/286 | 1 | 53 | 405 | 217 | 676 | 0,00000 | 0,00000 | 0,01854 | **0,98146** |
| **mSI_303** | **mSI_411** | 223/227 286/286 | 1 | 13 | 405 | 257 | 676 | 0,00005 | 0,00495 | 0,07143 | **0,92857** |
| **mSI_303** | **mSI_458** | 223/227 292/318 | 0 | 9 | 406 | 261 | 676 | 0,00024 | 0,01688 | 0,00000 | **1,00000** |
| **mSI_303** | **mSI_458** | 227/227 292/292 | 3 | 54 | 403 | 216 | 676 | 0,00000 | 0,00000 | 0,05267 | **0,94733** |
| **mSI_303** | **mSI_460** | 227/227 149/161 | 1 | 11 | 405 | 259 | 676 | 0,00028 | 0,01912 | 0,08334 | **0,91667** |
| **mSI_303** | **mSI_7** | 227/227 200/200 | 0 | 50 | 406 | 220 | 676 | 0,00000 | 0,00000 | 0,00000 | **1,00000** |
| **mSI_303** | **mSI_7** | 225/227 200/200 | 0 | 13 | 406 | 257 | 676 | 0,00001 | 0,00082 | 0,00000 | **1,00000** |
| **mSI_303** | **mSI_7** | 223/227 194/200 | 3 | 14 | 403 | 256 | 676 | 0,00053 | 0,03227 | 0,17647 | **0,82353** |
| **mSI_35** | **mSI_315** | 119/119 247/249 | 4 | 19 | 362 | 235 | 620 | 0,00004 | 0,00457 | 0,17392 | **0,82608** |
| **mSI_35** | **mSI_411** | 119/119 286/286 | 6 | 74 | 360 | 180 | 620 | 0,00000 | 0,00000 | 0,07500 | **0,92500** |
| **mSI_35** | **mSI_411** | 119/129 286/286 | 3 | 16 | 363 | 238 | 620 | 0,00015 | 0,01217 | 0,15791 | **0,84209** |
| **mSI_35** | **mSI_458** | 119/129 292/318 | 0 | 8 | 366 | 246 | 620 | 0,00074 | 0,03806 | 0,00000 | **1,00000** |
| **mSI_35** | **mSI_458** | 119/119 292/292 | 1 | 56 | 365 | 198 | 620 | 0,00000 | 0,00000 | 0,01758 | **0,98243** |
| **mSI_35** | **mSI_460** | 119/119 145/161 | 0 | 8 | 366 | 246 | 620 | 0,00074 | 0,03806 | 0,00000 | **1,00000** |
| **mSI_35** | **mSI_460** | 119/119 149/161 | 1 | 13 | 365 | 241 | 620 | 0,00007 | 0,00633 | 0,07144 | **0,92857** |
| **mSI_35** | **mSI_7** | 119/119 200/200 | 8 | 76 | 358 | 178 | 620 | 0,00000 | 0,00000 | 0,09524 | **0,90476** |
| **mSI_458** | **mSI_315** | 292/302 247/249 | 1 | 12 | 397 | 256 | 666 | 0,00013 | 0,01053 | 0,07693 | **0,92307** |
| **mSI_458** | **mSI_408** | 292/302 260/272 | 2 | 14 | 396 | 254 | 666 | 0,00012 | 0,00969 | 0,12508 | **0,87492** |
| **mSI_458** | **mSI_460** | 292/302 145/161 | 0 | 9 | 398 | 259 | 666 | 0,00026 | 0,01771 | 0,00000 | **1,00000** |
| **mSI_458** | **mSI_460** | 292/292 149/149 | 0 | 8 | 398 | 260 | 666 | 0,00065 | 0,03588 | 0,00000 | **1,00000** |
| **mSI_458** | **mSI_460** | 292/292 149/161 | 1 | 14 | 397 | 254 | 666 | 0,00002 | 0,00273 | 0,06668 | **0,93332** |
| **mSI_458** | **mSI_7** | 292/292 200/200 | 4 | 54 | 394 | 214 | 666 | 0,00000 | 0,00000 | 0,06897 | **0,93103** |
| **mSI_458** | **mSI_7** | 292/302 200/200 | 2 | 15 | 396 | 253 | 666 | 0,00005 | 0,00507 | 0,11776 | **0,88224** |
| **mSI_460** | **mSI_2** | 149/161 283/283 | 1 | 13 | 411 | 260 | 685 | 0,00005 | 0,00486 | 0,07143 | **0,92857** |
| **mSI_460** | **mSI_315** | 149/149 247/247 | 0 | 8 | 412 | 265 | 685 | 0,00060 | 0,03539 | 0,00000 | **1,00000** |
| **mSI_460** | **mSI_315** | 149/161 247/247 | 1 | 14 | 411 | 259 | 685 | 0,00002 | 0,00248 | 0,06668 | **0,93332** |
| **mSI_460** | **mSI_408** | 145/161 260/272 | 0 | 9 | 412 | 264 | 685 | 0,00023 | 0,01688 | 0,00000 | **1,00000** |
| **mSI_460** | **mSI_411** | 149/161 286/286 | 0 | 11 | 412 | 262 | 685 | 0,00004 | 0,00393 | 0,00000 | **1,00000** |
| **mSI_460** | **mSI_7** | 149/161 200/200 | 1 | 14 | 411 | 259 | 685 | 0,00002 | 0,00248 | 0,06668 | **0,93332** |
| **mSI_103** | **mSI_460** | 223/223 145/149 | 19 | 1 | 369 | 246 | 635 | 0,00080 | 0,04035 | **0,94995** | 0,05005 |
| **mSI_107** | **mSI_35** | 245/245 135/137 | 15 | 0 | 385 | 262 | 662 | 0,00068 | 0,03602 | **1,00000** | 0,00000 |
| **mSI_2** | **mSI_315** | 280/283 247/247 | 27 | 3 | 261 | 196 | 487 | 0,00020 | 0,01551 | **0,90000** | 0,10000 |
| **mSI_2** | **mSI_411** | 280/283 286/292 | 31 | 2 | 257 | 197 | 487 | 0,00001 | 0,00082 | **0,93937** | 0,06063 |
| **mSI_2** | **mSI_7** | 283/283 194/194 | 35 | 1 | 253 | 198 | 487 | 0,00000 | 0,00002 | **0,97222** | 0,02778 |
| **mSI_35** | **mSI_2** | 119/151 283/283 | 23 | 0 | 343 | 254 | 620 | 0,00000 | 0,00073 | **1,00000** | 0,00000 |
| **mSI_35** | **mSI_315** | 135/137 247/247 | 15 | 0 | 351 | 254 | 620 | 0,00065 | 0,03588 | **1,00000** | 0,00000 |
| **mSI_35** | **mSI_408** | 135/137 260/272 | 15 | 0 | 351 | 254 | 620 | 0,00065 | 0,03588 | **1,00000** | 0,00000 |
| **mSI_458** | **mSI_408** | 292/302 272/272 | 20 | 1 | 378 | 267 | 666 | 0,00039 | 0,02514 | **0,95231** | 0,04769 |
| **mSI_460** | **mSI_408** | 145/161 272/272 | 15 | 0 | 397 | 273 | 685 | 0,00067 | 0,03588 | **1,00000** | 0,00000 |

Significant predictions for self-compatible/incompatible plants, according to specific genotype presence or combinations of two genotypes were evidenced by a Fisher´s Exact Test. Only the predictions made with combinations of two genotypes, improved compared to those made with one genotype, are reported. Probabilities associated to the tests were adjusted for multiple testing, using the QVALUE software. The probabilities of being self-incompatible, given the individuals have (ProbSIYs) or not have (ProbSINo) the specified genotype was computed from a logistic regression. S_0 and S_1: number of individuals having the genotype(s) and being self-incompatible (0) or self-compatible (1); N_0 and N_1: number of individuals not having the genotype(s) and being self-incompatible (0) or self-compatible (1).

**Table S7. Significant predictions of self-compatibility/incompatibility based on allele presence.**

| | | | | | Number of plants in each category | | | | | | Probability being SC | | |
|---|---|---|---|---|---|---|---|---|---|---|---|---|---|
| marker | CH | Allele | XP2_FISH | q-value | 0 copy-SI | 0 copy-SC | 1 copy-SI | 1 copy-SC | 2 copies-SI | 2 copies-SC | 0 copy | 1 copy | 2 copies |
| mSI_103 | CH1 | 223 | 4,3E-03 | 1,6E-02 | 202 | 93 | 151 | 143 | 76 | 45 | 0,354 | 0,409 | 0,466 |
| mSI_103 | CH1 | 225 | 0,0E+00 | 0,0E+00 | 339 | 257 | 73 | 23 | 17 | 1 | 0,432 | 0,224 | 0,098 |
| mSI_107 | CH1 | 237 | 4,7E-03 | 1,6E-02 | 422 | 281 | 2 | 0 | 5 | 0 | 0,400 | 0,000 | 0,000 |
| mSI_107 | CH1 | 239 | 0,0E+00 | 0,0E+00 | 368 | 192 | 44 | 77 | 17 | 12 | 0,356 | 0,514 | 0,669 |
| mSI_107 | CH1 | 241 | 2,2E-04 | 1,2E-03 | 403 | 276 | 17 | 5 | 9 | 0 | 0,407 | 0,176 | 0,062 |
| mSI_107 | CH1 | 243 | 1,1E-04 | 6,5E-04 | 338 | 247 | 61 | 26 | 30 | 8 | 0,422 | 0,303 | 0,206 |
| mSI_107 | CH1 | 245 | 2,8E-03 | 1,1E-02 | 173 | 71 | 100 | 107 | 156 | 103 | 0,343 | 0,394 | 0,447 |
| mSI_2 | CH4 | 292 | 3,8E-03 | 1,4E-02 | 422 | 281 | 3 | 0 | 4 | 0 | 0,400 | 0,000 | 0,000 |
| mSI_303 | CH4 | 211 | 4,9E-03 | 1,6E-02 | 395 | 268 | 20 | 12 | 14 | 1 | 0,407 | 0,275 | 0,173 |
| mSI_303 | CH4 | 221 | 1,9E-04 | 1,0E-03 | 389 | 271 | 31 | 10 | 9 | 0 | 0,412 | 0,206 | 0,087 |
| mSI_303 | CH4 | 227 | 0,0E+00 | 0,0E+00 | 256 | 65 | 156 | 159 | 17 | 57 | 0,207 | 0,496 | 0,788 |
| mSI_303 | CH4 | 231 | 0,0E+00 | 0,0E+00 | 347 | 264 | 57 | 17 | 25 | 0 | 0,435 | 0,187 | 0,064 |
| mSI_303 | CH4 | 233 | 1,4E-04 | 8,0E-04 | 297 | 227 | 109 | 47 | 23 | 7 | 0,432 | 0,310 | 0,210 |
| mSI_303 | CH4 | 235 | 1,3E-02 | 3,8E-02 | 413 | 279 | 16 | 2 | 0 | 0 | 0,403 | 0,111 | . |
| mSI_303 | CH4 | 237 | 3,5E-04 | 1,7E-03 | 399 | 275 | 24 | 6 | 6 | 0 | 0,409 | 0,176 | 0,062 |
| mSI_35 | CH4 | 119 | 0,0E+00 | 0,0E+00 | 184 | 52 | 217 | 143 | 28 | 86 | 0,195 | 0,430 | 0,702 |
| mSI_35 | CH4 | 125 | 2,1E-03 | 8,8E-03 | 413 | 280 | 16 | 1 | 0 | 0 | 0,404 | 0,059 | . |
| mSI_35 | CH4 | 135 | 0,0E+00 | 0,0E+00 | 345 | 257 | 69 | 23 | 15 | 1 | 0,428 | 0,233 | 0,110 |
| mSI_35 | CH4 | 137 | 7,0E-05 | 4,7E-04 | 328 | 247 | 99 | 33 | 2 | 1 | 0,429 | 0,259 | 0,140 |
| mSI_35 | CH4 | 151 | 1,0E-05 | 9,9E-05 | 361 | 266 | 68 | 15 | 0 | 0 | 0,424 | 0,181 | . |
| mSI_411 | CH4 | 284 | 7,0E-05 | 4,7E-04 | 315 | 239 | 98 | 39 | 16 | 3 | 0,432 | 0,282 | 0,168 |
| mSI_411 | CH4 | 286 | 0,0E+00 | 0,0E+00 | 110 | 44 | 205 | 113 | 114 | 124 | 0,263 | 0,377 | 0,506 |
| mSI_411 | CH4 | 292 | 7,1E-03 | 2,2E-02 | 207 | 165 | 201 | 111 | 21 | 5 | 0,448 | 0,345 | 0,255 |
| mSI_458 | CH4 | 292 | 0,0E+00 | 0,0E+00 | 250 | 62 | 142 | 138 | 37 | 81 | 0,214 | 0,459 | 0,726 |
| mSI_458 | CH4 | 296 | 0,0E+00 | 0,0E+00 | 392 | 281 | 32 | 0 | 5 | 0 | 0,418 | 0,000 | 0,000 |
| mSI_458 | CH4 | 302 | 1,0E-04 | 6,2E-04 | 362 | 258 | 45 | 19 | 22 | 4 | 0,417 | 0,280 | 0,175 |
| mSI_458 | CH4 | 304 | 2,7E-03 | 1,1E-02 | 364 | 250 | 35 | 25 | 30 | 6 | 0,412 | 0,323 | 0,245 |
| mSI_458 | CH4 | 306 | 0,0E+00 | 0,0E+00 | 314 | 244 | 89 | 35 | 26 | 2 | 0,440 | 0,256 | 0,130 |
| mSI_458 | CH4 | 312 | 2,0E-03 | 8,8E-03 | 397 | 271 | 23 | 10 | 9 | 0 | 0,407 | 0,236 | 0,122 |
| mSI_458 | CH4 | 318 | 1,5E-03 | 6,9E-03 | 422 | 263 | 7 | 18 | 0 | 0 | 0,384 | 0,720 | . |
| mSI_460 | CH4 | 131 | 7,0E-05 | 4,7E-04 | 412 | 281 | 15 | 0 | 2 | 0 | 0,405 | 0,000 | 0,000 |
| mSI_460 | CH4 | 136 | 2,2E-03 | 8,8E-03 | 400 | 276 | 29 | 5 | 0 | 0 | 0,408 | 0,147 | . |
| mSI_460 | CH4 | 138 | 4,7E-03 | 1,6E-02 | 392 | 231 | 30 | 49 | 7 | 1 | 0,378 | 0,514 | 0,649 |
| mSI_460 | CH4 | 139 | 1,3E-03 | 6,4E-03 | 415 | 281 | 14 | 0 | 0 | 0 | 0,404 | 0,000 | . |
| mSI_460 | CH4 | 145 | 7,0E-05 | 4,7E-04 | 290 | 226 | 129 | 54 | 10 | 1 | 0,440 | 0,286 | 0,169 |
| mSI_460 | CH4 | 146 | 4,0E-05 | 3,3E-04 | 370 | 267 | 55 | 14 | 4 | 0 | 0,420 | 0,194 | 0,074 |
| mSI_460 | CH4 | 149 | 1,0E-04 | 6,2E-04 | 362 | 208 | 65 | 63 | 2 | 10 | 0,362 | 0,521 | 0,677 |
| mSI_460 | CH4 | 155 | 0,0E+00 | 0,0E+00 | 374 | 280 | 52 | 1 | 3 | 0 | 0,428 | 0,019 | 0,000 |
| mSI_460 | CH4 | 161 | 0,0E+00 | 0,0E+00 | 288 | 119 | 141 | 118 | 0 | 44 | 0,268 | 0,531 | 1,000 |
| mSI_7 | CH4 | 178 | 3,0E-05 | 2,6E-04 | 381 | 272 | 42 | 9 | 6 | 0 | 0,417 | 0,164 | 0,051 |
| mSI_7 | CH4 | 194 | 2,0E-05 | 1,9E-04 | 139 | 128 | 183 | 117 | 107 | 36 | 0,489 | 0,373 | 0,270 |
| mSI_7 | CH4 | 197 | 0,0E+00 | 0,0E+00 | 381 | 277 | 43 | 4 | 5 | 0 | 0,421 | 0,083 | 0,011 |
| mSI_7 | CH4 | 200 | 0,0E+00 | 0,0E+00 | 200 | 73 | 180 | 120 | 49 | 88 | 0,254 | 0,425 | 0,615 |

Significant allele effect evidenced by a Fisher´s Exact Test. Probabilities associated to the tests were adjusted for multiple testing, using the QVALUE software. The effect of increasing the allele dose (0, 1, 2 copies) and the probability of being self-compatible was evaluated by fitting a logistic regression to the data.
