# Supplementary figures and images for: Deciphering the Theobroma cacao self-incompatibility system: from genomics to diagnostic markers for self-compatibility
Source: J Exp Bot. 2017 Oct 7;68(17):4775–90. doi: 10.1093/jxb/erx293 (PMC5853246; doi:10.1093/jxb/erx293)

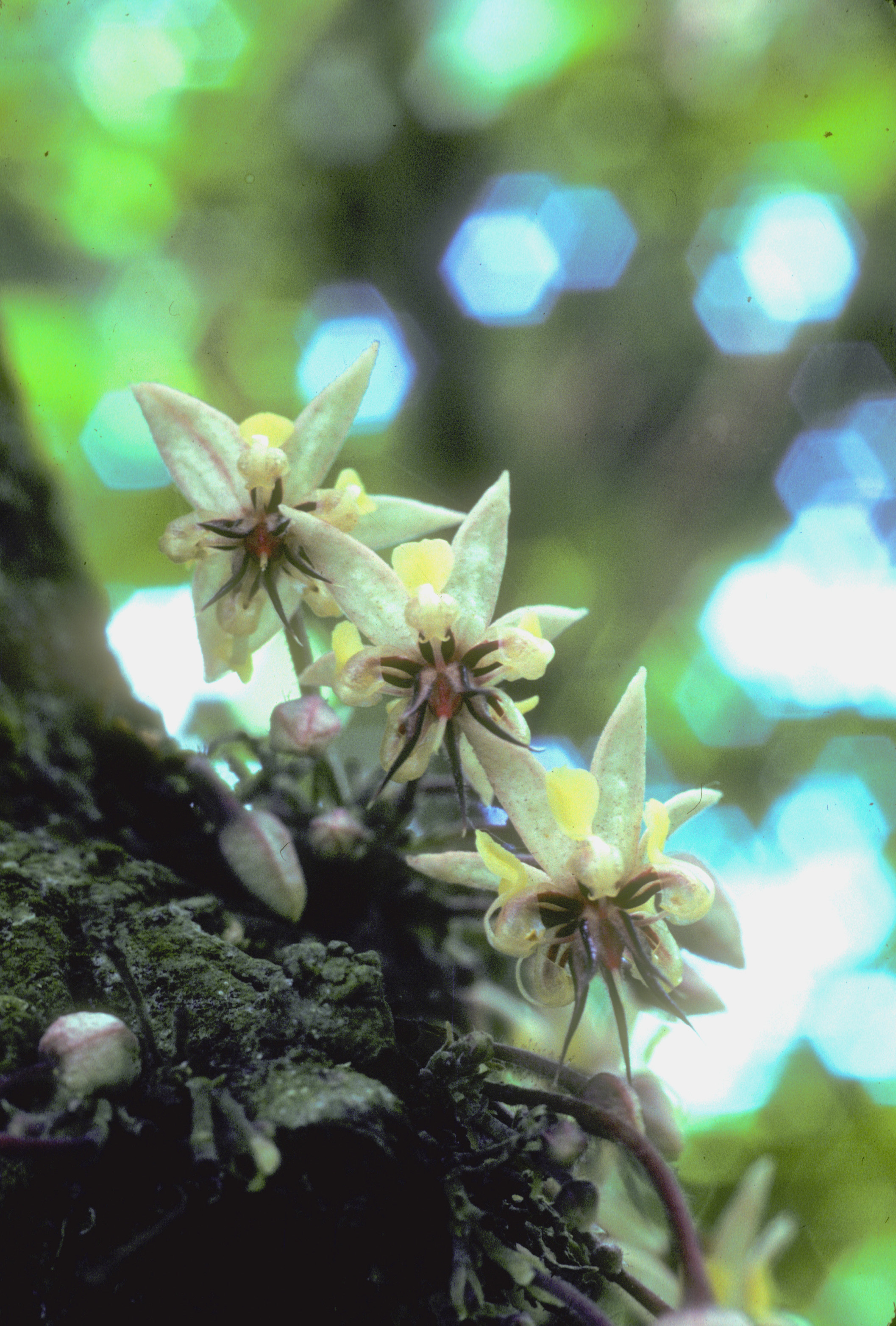

Supplement: Supplementary File [file erx293_suppl_supplementary_file.jpeg]
